# Supplementary material for: Early-life gut microbiota assembly patterns are conserved between laboratory and wild mice
Source: Commun Biol. 2024 Nov 7;7:1456. doi: 10.1038/s42003-024-07039-y (PMC11543677; doi:10.1038/s42003-024-07039-y)
Supplement: Supplementary file 1 — Supplementary Information [file 42003_2024_7039_MOESM1_ESM.pdf]

Supplementary data

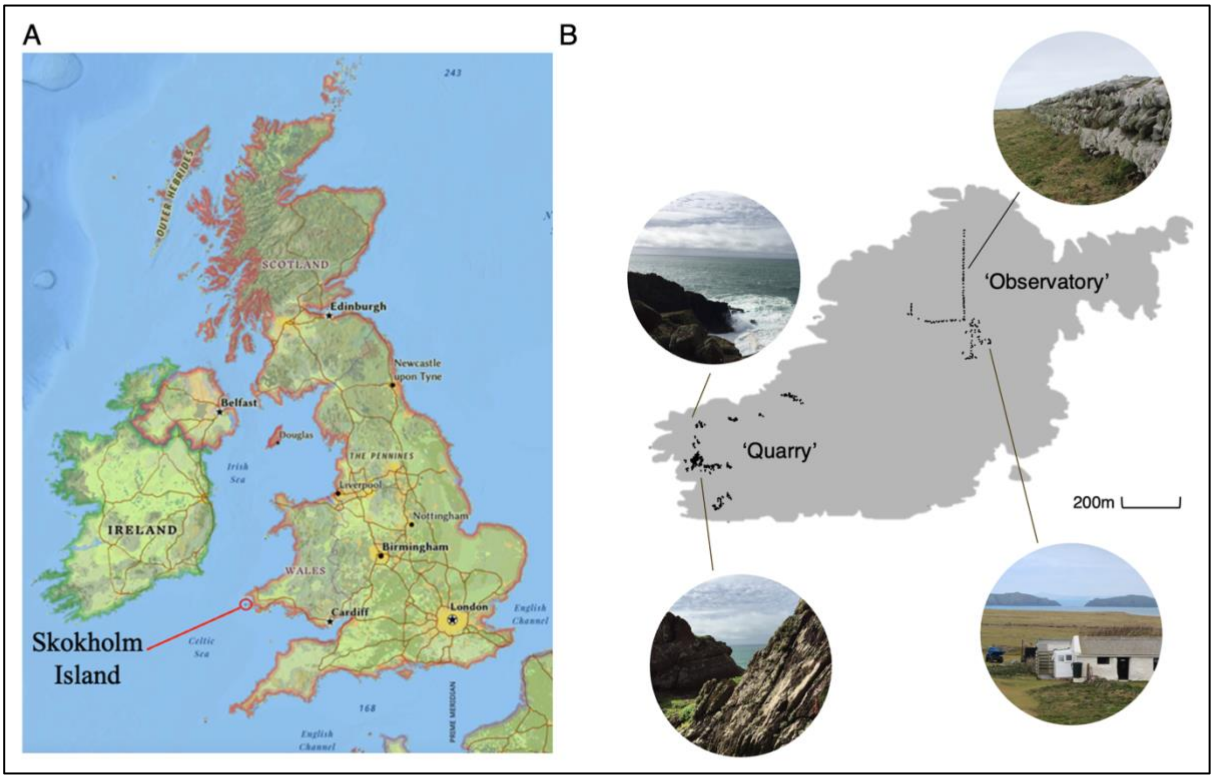

Supplementary Figure 1. (**A**) Skokholm Island is located 4 km off the coast of Pembrokeshire in south-west Wales, UK. (**B**) Two wild house mouse sampling sites, 'Observatory' and 'Quarry', on Skokholm Island. 150 trapping points (black circles) were distributed at each site. Map was generated using ArcGIS. Photos: Eveliina Hanski.

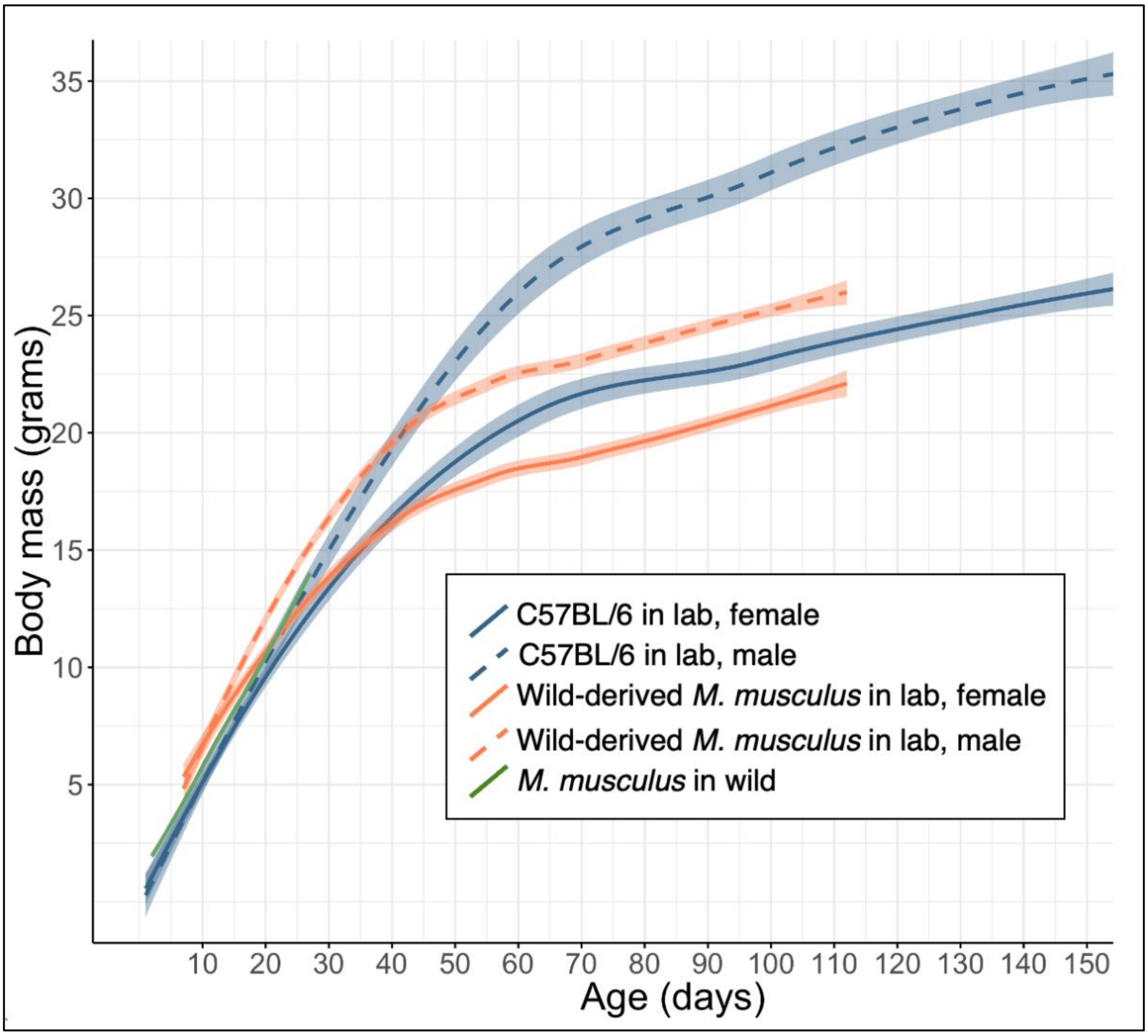

Supplementary Figure 2. Age–body mass relationship in house mouse. *Blue:* C57BL/6NCrl and C57BL/6J (*Mus musculus*) data has been reproduced from Figure 3 in Spangenberg et al (2014)[1] and The Jackson Laboratory website[2], respectively. *Orange:* Wild-derived *M. musculus* (Gough Island, home to the largest wild house mice recorded; mice born in laboratory) data has been reproduced from Figure 3 in Gray et al (2015)[3]. *Green:* Wild *M. musculus* data has been reproduced from Figure 5 in Ferrari et al (2015)[4], and depicts the body masses of young wild mice of known age inhabiting a barn near Zurich, Switzerland. Line type indicates sex: *solid* = female, *dashed* = male.

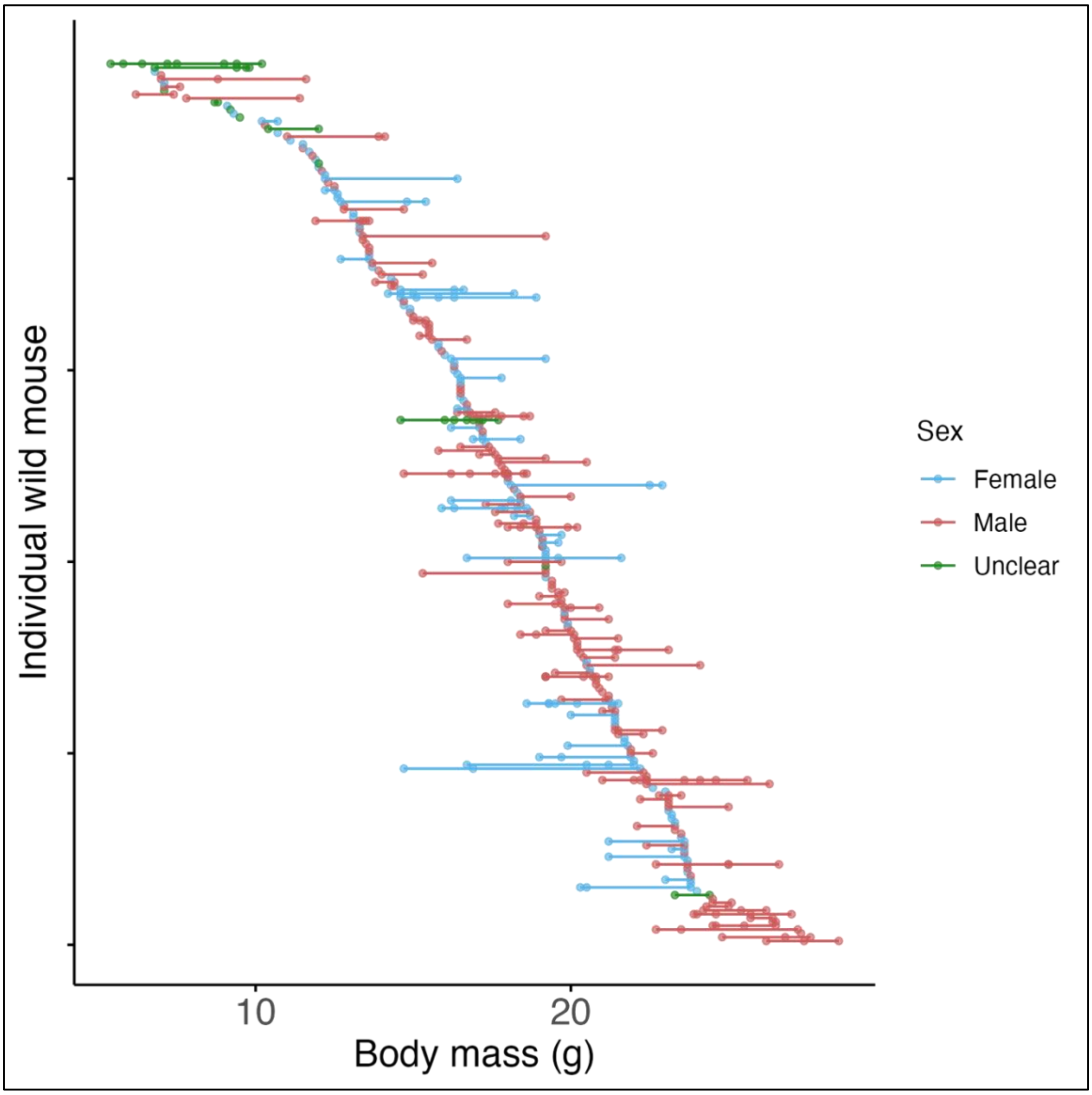

Supplementary Figure 3. Sampling history of 230 individual wild mice included in the analyses (1–10 samples per individual, total 433 samples).

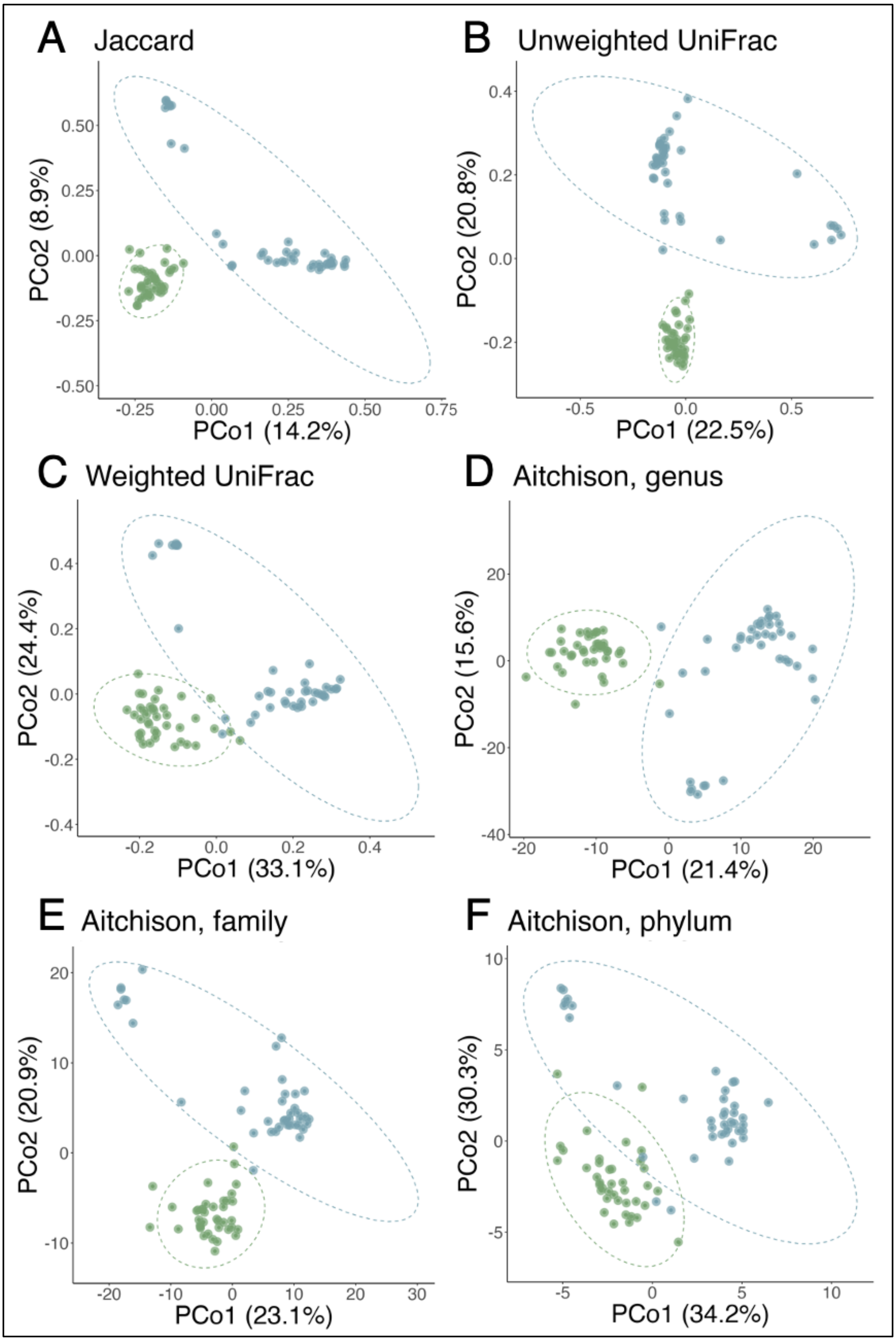

Supplementary Figure 4. Principal coordinates analysis (PCoA) of 78 faecal samples from 39 laboratory and 39 wild mice using (**A**) Jaccard, (**B**) unweighted UniFrac, (**C**) weighted and (**D**–**F**) Aitchison distances at (**A–C**) amplicon sequence variant (ASV), (**D**) genus, (**E**) family, or (**F**) phylum level. Colour indicates sample source (*blue* = laboratory, *green* = wild).

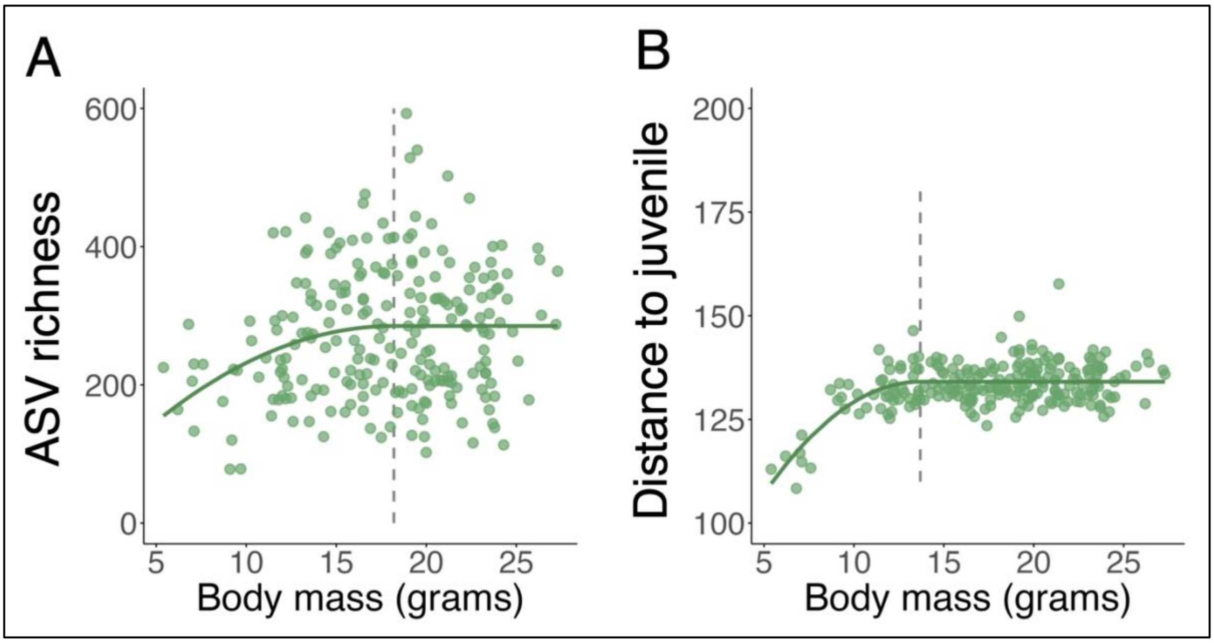

Supplementary Figure 5. Taxon-independent trends in wild house mouse gut microbiota using body mass as a proxy of age. Only one sample per individual (first available sample) is included (*n*=230). Body mass ranges 5.4–27.3g. (**A**) Quadratic plateau model on asymptotic ASV richness. R^2=0.043, critical point of inflexion=18.2g. (**B)** Quadratic plateau model on the relationship between Aitchison distance to reference juvenile microbiota and body mass. Reference juvenile microbiota was measured by taking the mean of all taxon abundances in wild mice ≤7.9g of body mass (*n*=7), which is the estimated equivalent of 14 days of age in wild mice. R^2=0.327, critical point of inflexion=13.7g. Vertical lines indicate critical points of inflection.

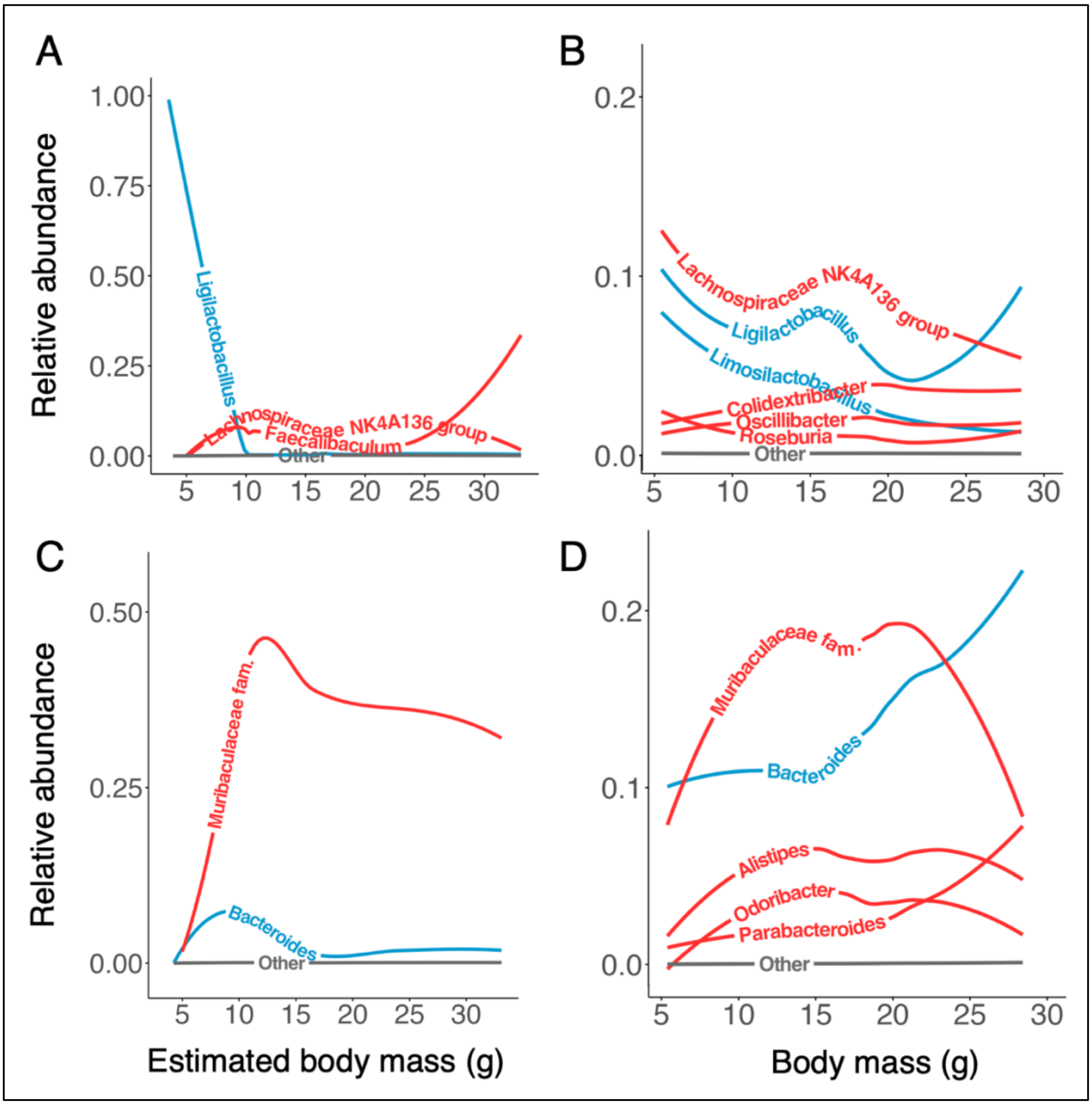

Supplementary Figure 6. Relative abundance of genera from (**A–B**) Firmicutes and (**C–D**) Bacteroidota, in (**A, C**) laboratory and (**B, D**) wild mice. Relative abundances are measured from the whole microbiota, rather than within phylum. Lines are locally estimated scatterplot smoothing (LOESS) lines. Confidence intervals are not plotted for easier interpretation. Lines are coloured by genus aerotolerance (*red* = obligate anaerobes, *blue* = aerotolerant, *grey* = unknown aerotolerance). Note the variable y-axes scales to aid visibility of taxa with low abundance.

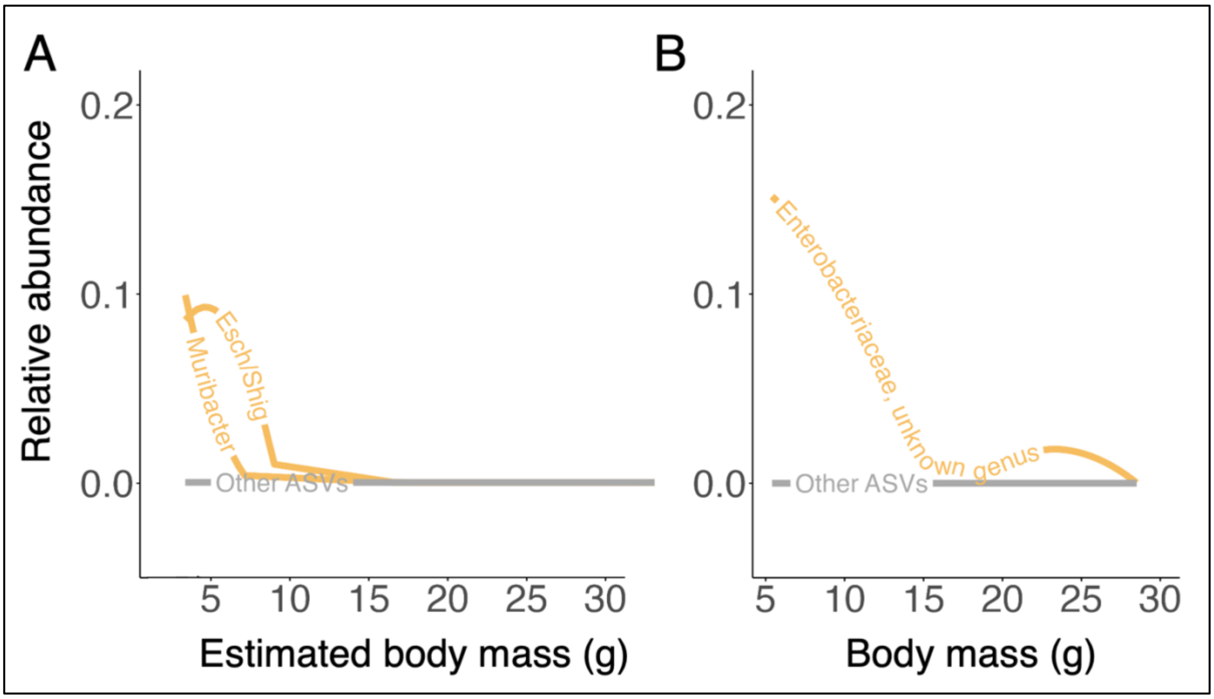

Supplementary Figure 7. Relative abundance of ASVs assigned to the phylum Proteobacteria in (**A**) lab and (**B**) wild mouse gut microbiota. *Esch/Shig* = Escherichia/Shigella.

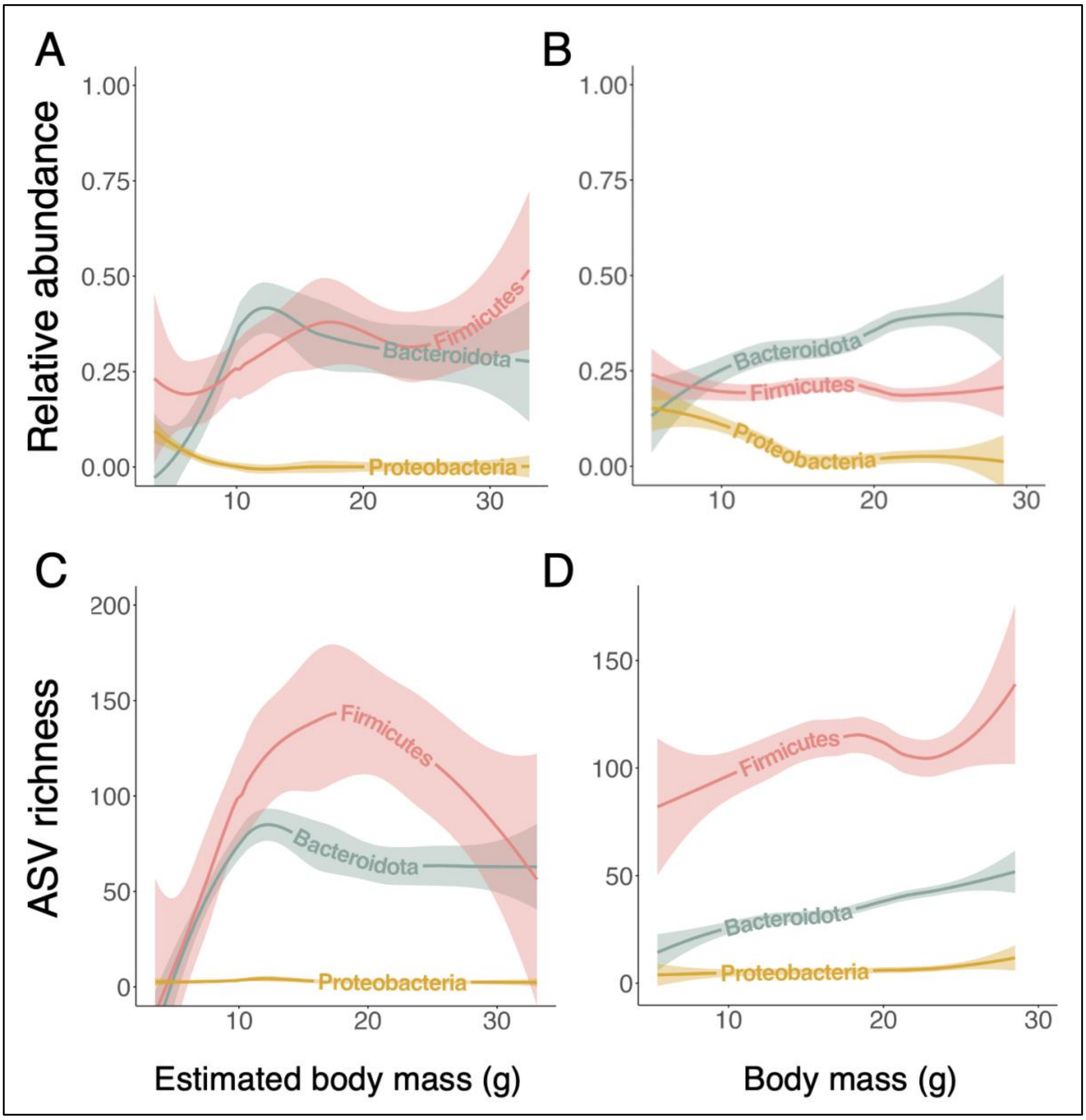

Supplementary Figure 8. Dynamics in bacterial phyla based on amplicon sequence variants (ASVs) that were only detected in (**A**, **C**) lab or (**B**, **D**) wild mice. (**A–B**) Relative abundance of Firmicutes, Bacteroidota, and Proteobacteria. (**C–D**) ASV richness (total count of unique ASVs) in Firmicutes, Bacteroidota, and Proteobacteria. Lines are locally estimated scatterplot smoothing (LOESS) lines with 95% confidence interval bands.

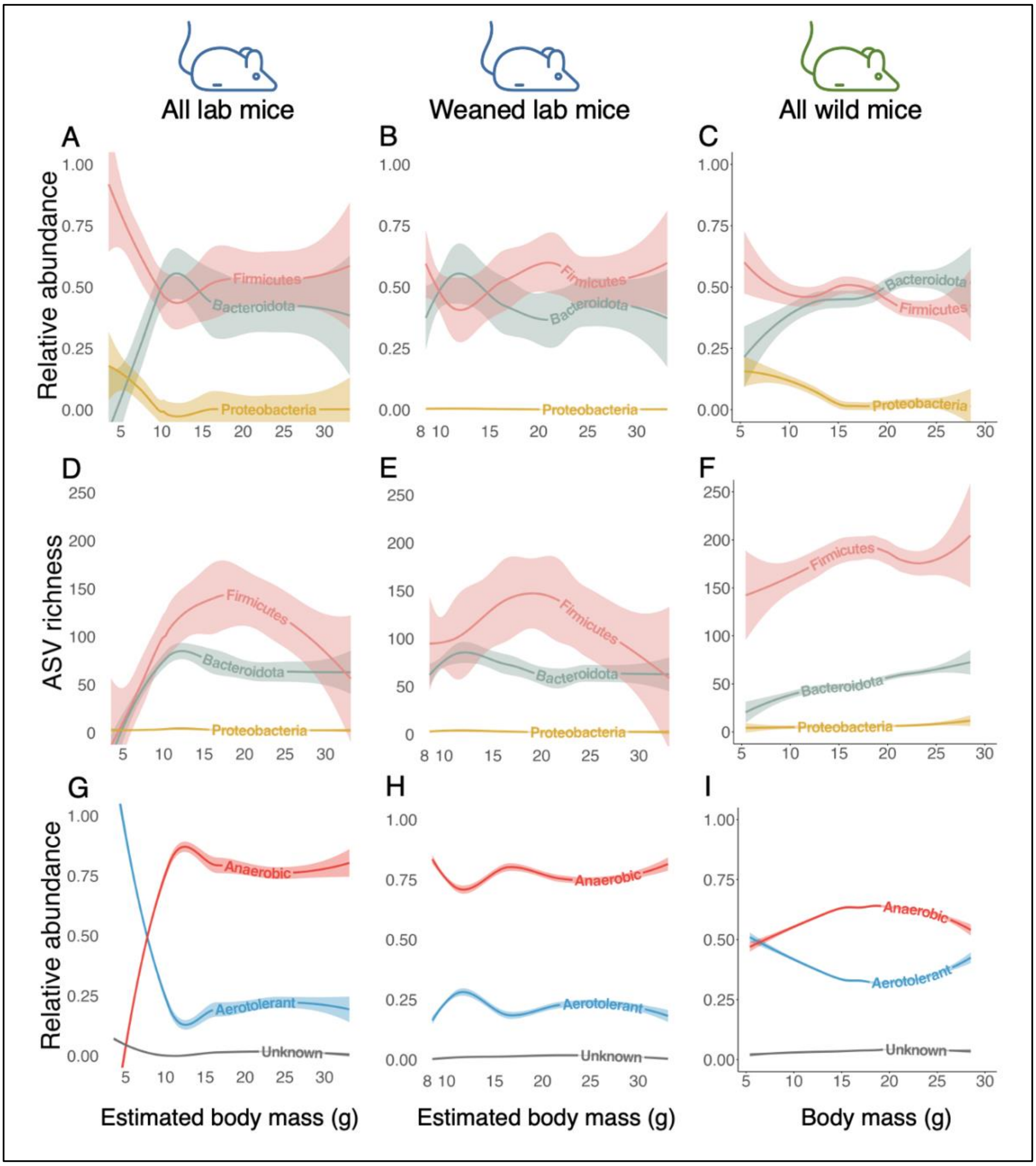

Supplementary Figure 9. Age-related gut microbial dynamics in (**A, B, D, E, G, H**) lab and (**C, F, I**) wild mice. Lab mouse data is limited to samples from weaned individuals in middle panels (**B, E, H**). Relative abundance of (**A–C**) predominant phyla (abundances were measured from all taxa), (**D–F**) ASV richness (total count of unique ASVs) in predominant phyla, and (**G–I**) aerotolerant and obligate anaerobic bacteria across (estimated) body mass. Lines are locally estimated scatterplot smoothing (LOESS) lines with 95% confidence interval bands. *Anaerobic* = obligate anaerobes, *aerotolerant* = everything else with known aerotolerance, *unknown* = bacteria with unknown aerotolerance.

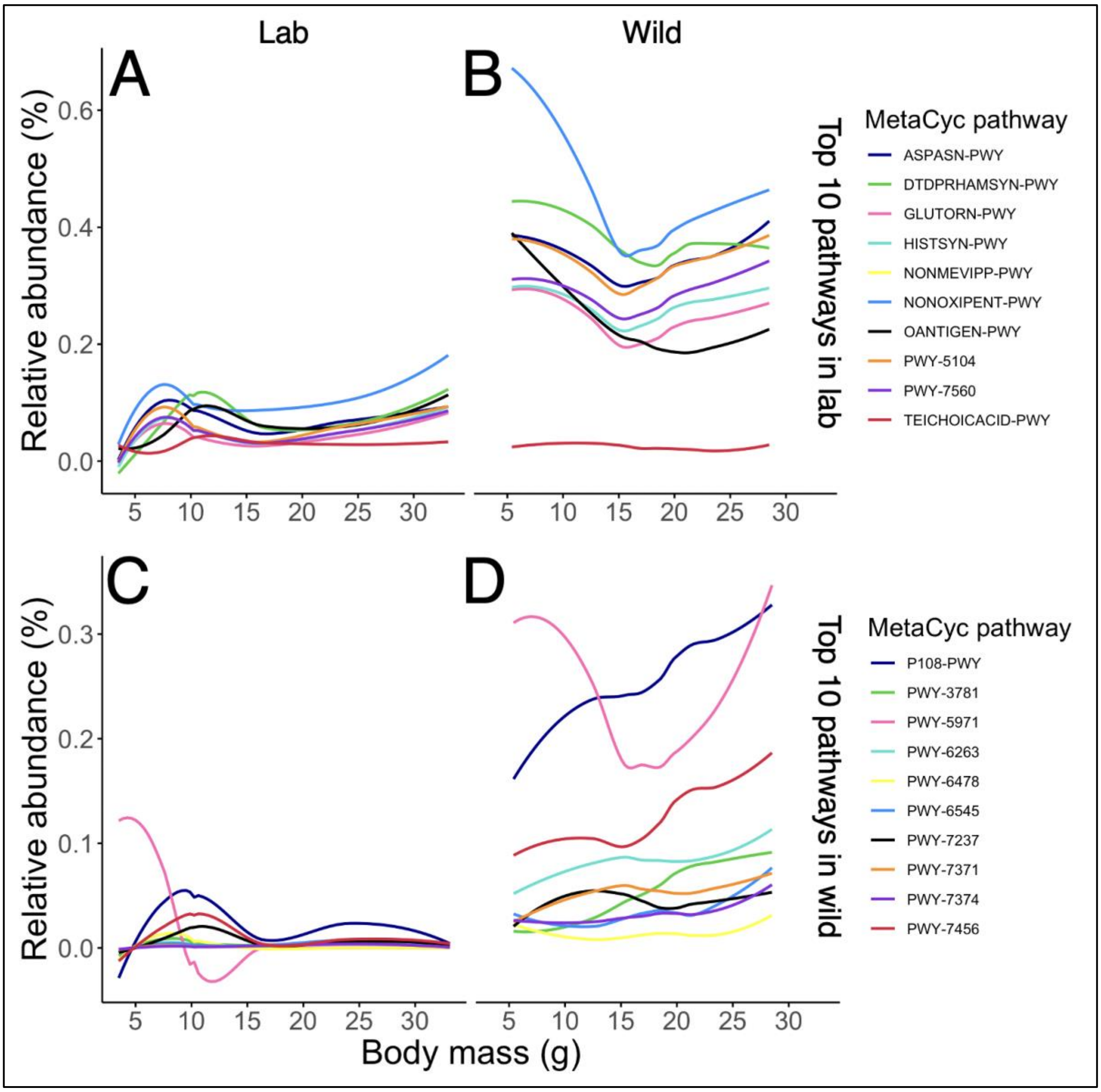

Supplementary Figure 10. Relationship between body mass and the relative abundance of MetaCyc pathways with the largest fold-change between juvenile (weighing ≤7g) and adult mice (weighing >20g). Panels **A** and **B** show relative abundance of the 10 pathways with the greatest age-related fold change in lab mice, plotted in (**A**) lab and (**B**) wild mice. Panels **C** and **D** show relative abundance of the 10 pathways with the greatest age-related fold change in wild mice, plotted in (**C**) lab or (**D**) wild mice. Lines are locally estimated scatterplot smoothing (LOESS) lines. Full MetaCyc pathways names are provided in Table S2.

Supplementary Table 1. List of pathogens excluded in the SPF-facility where laboratory mice sampled in this study were housed.

Serology
1. MHV Mouse Hepatitis Virus
2. EDIM Epizootic Diarrhoea of Infant Mice
3. MNV Mouse Norovirus
4. MVM Minute Virus of Mice
5. MPV Mouse Parvovirus
6. GDVII TMEV
7. LCMV Lymphocytic Choriomeningitis Virus
8. MAD1 Mouse Adenovirus 1
9. MAD2 Mouse Adenovirus 2
10. ECTRO Ectromelia Virus
11. PVM Pneumonia Virus of Mice
12. REO III Reo Virus Type 3
13. SEND Sendai Virus
14. CPIL Clostridium piliforme
15. MPUL Mycoplasma species
16. PMUR Pneumocystis murina
Parasitology
1. Giardia
2. Spironucleus
3. Chilomastix Sp
4. Entamoeba muris
5. Enteromonas Sp
6. Trichomonas Sp
7. Tetratrichomonas Sp
8. Aspiculuris tetraptera
9. Syphacia obvelata
10. Faecal Ova
11. Arthropods
Bacteriology
1. Helicobacter species
2. Pasteurella pneumotropica (Heyl)
3. Pasteurella pneumotropica (Jawetz)
4. Streptococcus Beta Haemolytic (NOT Group D)
5. Streptococcus pneumoniae
6. Citrobacter rodentium
7. Corynebacterium kutscheri
8. Salmonella species
9. Streptobacillus moniliformis

Supplementary Table 2.

MetaCyc pathways and their abbreviations.

MetaCyc pathway abbreviation	***MetaCyc pathway name***
ASPASN-PWY	superpathway of L-aspartate and L-asparagine biosynthesis
DTDPRHAMSYN-PWY	dTDP-L-rhamnose biosynthesis
GLUTORN-PWY	L-ornithine biosynthesis I
HISTSYN-PWY	L-histidine biosynthesis
NONMEVIPP-PWY	methylerythritol phosphate pathway I
NONOXIPENT-PWY	pentose phosphate pathway (non-oxidative branch) I
OANTIGEN-PWY	O-antigen building blocks biosynthesis (E. coli)
PWY-5104	L-isoleucine biosynthesis IV
PWY-7560	methylerythritol phosphate pathway II
TEICHOICACID-PWY	poly(glycerol phosphate) wall teichoic acid biosynthesis
P108-PWY	pyruvate fermentation to propanoate I
PWY-3781	aerobic respiration I (cytochrome c)
PWY-5971	palmitate biosynthesis II (type II fatty acid synthase)
PWY-6263	superpathway of menaquinol-8 biosynthesis II
PWY-6478	GDP-D-*glycero*-α-D-*manno*-heptose biosynthesis
PWY-6545	pyrimidine deoxyribonucleotides *de novo*biosynthesis III
PWY-7237	*myo*-, *chiro*- and *scyllo*-inositol degradation
PWY-7371	5,8-dihydroxy-2-naphthoate biosynthesis II
PWY-7374	5,8-dihydroxy-2-naphthoate biosynthesis I
PWY-7456	β-(1,4)-mannan degradation

Supplementary references

1. Spangenberg, E., Wallenbeck, A., Eklöf, A.-C., Carlstedt-Duke, J. & Tjäder, S. Housing breeding mice in three different IVC systems: maternal performance and pup development. *Lab Anim* 48, 193–206 (2014).
2. Body Weight Information for C57BL/6J | The Jackson Laboratory. https://www.jax.org/jax-mice-and-services/strain-data-sheet-pages/body-weight-chart-000664.
3. Gray, M. M. *et al.* Genetics of Rapid and Extreme Size Evolution in Island Mice. *Genetics* 201, 213–228 (2015).
4. Ferrari, M., Lindholm, A. K. & König, B. The risk of exploitation during communal nursing in house mice, Mus musculus domesticus. *Anim Behav* 110, 133–143 (2015).
