# Supplementary material for: Early-life gut microbiota assembly patterns are conserved between laboratory and wild mice
Source: Commun Biol. 2024 Nov 7;7:1456. doi: 10.1038/s42003-024-07039-y (PMC11543677; doi:10.1038/s42003-024-07039-y)
Supplement: Supplementary file 2 — Description of Additional Supplementary Files [file 42003_2024_7039_MOESM2_ESM.pdf]

Description of Additional Supplementary Files

File name: Supplementary Data 1
Description: Information of wild and laboratory mouse samples included in the study.

File name: Supplementary Data 2
Description: Aerotolerance ability of bacterial genera detected across laboratory and wild mice.

File name: Supplementary Data 3-6
Description: Source data for Figures 1–4.

:
