# Supplementary material for: Early-life gut microbiota assembly patterns are conserved between laboratory and wild mice
Source: Commun Biol. 2024 Nov 7;7:1456. doi: 10.1038/s42003-024-07039-y (PMC11543677; doi:10.1038/s42003-024-07039-y)
Supplement: Supplementary file 4 — Supplemenetary Data 2 [file 42003_2024_7039_MOESM4_ESM.pdf]

Supplementary Table 3. Aerotolerance of bacterial genera detected across laboratory and wild mice.

Bergey's Manual of Systematics of Archae and Bacteria alongside additional references (listed below) were used to determine aerotolerance (A = aerotolerant, OA = obligate anaerobe). Bacteria were classified as obligate anaerobes only when explicitly listed as obligate anaerobes. Aerotolerance was determined based on genus listed in Genus. If multiple genera were assigned for a given ASV (e.g., "Methylobacterium-Methylorubrum", genus based on which aerotolerance was determined is listed in Comments. If genus-level information was not available, family-level information was inspected and used if all genera from a given family were stated to have same aerotolerance (indicated in Comments as 'Based on -ceae').

Aerotolerance_category	
A	Aerotolerant
AN	Anaerobic
N/A	Not applicable

Family	Genus	Aerotolerance_category	Reference	Comments
[Clostridium] methylpentosum group	Other	N/A	NA	
[Clostridium] methylpentosum group	Other	N/A	Bergey's manual	Varies within family
[Eubacterium] coprostanoligenes group	Other	AN	Bergey's manual	Based on Eubacterium
[Eubacterium] coprostanoligenes group	Other	N/A	NA	
67-14	Other	N/A	NA	
A4b	Other	N/A	NA	
Abditibacteriaceae	Abditibacterium	N/A	NA	
Acetobacteraceae	Acetobacter	A	Bergey's Manual	
Acetobacteraceae	Acidicaldus	A	Johnson et al., 2006	
Acetobacteraceae	Acidiphilium	A	Bergey's Manual	
Acetobacteraceae	Acidisoma	A	Bergey's Manual	
Acetobacteraceae	Endobacter	A	Bergey's Manual	Based on Acetobacteraceae
Acetobacteraceae	Gluconobacter	A	Bergey's Manual	
Acetobacteraceae	Other	A	Bergey's Manual	Based on Acetobacteraceae
Acetobacteraceae	Other	A	Bergey's manual	
Acetobacteraceae	Rhodovastum	A	Bergey's Manual	Based on Acetobacteraceae
Acetobacteraceae	Roseomonas	A	Bergey's Manual	
Acholeplasmataceae	Anaeroplasma	AN	Bergey's Manual	
Acholeplasmataceae	Other	N/A	NA	
Acholeplasmataceae	Other	N/A		
Acidaminococcaceae	Phascolarctobacterium	AN	Bergey's Manual	
Acidobacteriaceae (Subgroup 1)	Edaphobacter	A	Bergey's Manual	
Acidothermaceae	Acidothermus	A	Bergey's Manual	
Actinomycetaceae	Actinomyces	A	Bergey's Manual	
Aerococcaceae	Facklamia	A	Bergey's Manual	
Aerococcaceae	Other	A	Bergey's Manual	Based on Aerococcaceae
Aerococcaceae	Other	A	Bergey's manual	
Aeromonadaceae	Oceanisphaera	A	Bergey's Manual	Based on Aeromonadaceae
Akkermansiaceae	Akkermansia	AN	Bergey's Manual	
AKYG1722	Other	N/A	NA	
Alcaligenaceae	Achromobacter	A	Bergey's Manual	
Alcaligenaceae	Alcaligenes	A	Bergey's Manual	Based on Alcaligenaceae
Alcaligenaceae	Candidimonas	A	Bergey's Manual	
Alcaligenaceae	Eoetvoesia	A	Bergey's Manual	Based on Alcaligenaceae
Alcaligenaceae	Other	A	Bergey's Manual	Based on Alcaligenaceae

Alcaligenaceae	Other	A	Bergey's manual	
Alcaligenaceae	Paenalcaligenes	A	Bergey's Manual	
Alcaligenaceae	Verticiella	A	Vandamme et al. 2015	Based on Verticia
Alicyclobacillaceae	Tumebacillus	A	Bergey's Manual	
Amoebophilaceae	Candidatus Cardinium	N/A		
Anaerofustaceae	Anaerofustis	AN	Bergey's Manual	
Anaerovoracaceae	[Eubacterium] brachy gro	AN	Bergey's Manual	Based on Eubacterium
Anaerovoracaceae	[Eubacterium] nodatum g	AN	Bergey's Manual	Based on Eubacterium
Anaerovoracaceae	Anaerovorax	AN	Bergey's Manual	
Anaerovoracaceae	Family XIII AD3011 group	A	Bergey's Manual	Based on Anaerovorax
Anaerovoracaceae	Family XIII UCG-001	A	Bergey's Manual	Based on Anaerovorax
Anaerovoracaceae	Other	N/A	NA	
Anaerovoracaceae	Other	N/A		
Anaplasmataceae	Wolbachia	N/A		
Atopobiaceae	Coriobacteriaceae UCG-0	A	Bergey's Manual	Based on Atopobiaceae
Atopobiaceae	Coriobacteriaceae UCG-0	A	Bergey's Manual	Based on Atopobiaceae
Azospirillaceae	Skermanella	A	Bergey's Manual	
Bacillaceae	Allobacillus	N/A		
Bacillaceae	Bacillus	A	Bergey's Manual	
Bacillaceae	Falsibacillus	N/A		
Bacillaceae	Microaerobacter	N/A		
Bacillaceae	Natronobacillus	N/A		
Bacillaceae	Other	A	Bergey's manual	
Bacillaceae	Other	N/A	NA	
Bacteroidaceae	Bacteroides	A	Bergey's Manual	
Bacteroidales RF16 group	Other	A	Bergey's Manual	Based on Bacteroidales RF16 group
Bacteroidales RF16 group	Other	N/A		
Barnesiellaceae	Barnesiella	AN	Bergey's Manual	
Barnesiellaceae	Coprobacter	N/A		
Barnesiellaceae	Other	N/A	NA	
Barnesiellaceae	Other	N/A		
Bdellovibrionaceae	Bdellovibrio	A	Bergey's Manual	
Beijerinckiaceae	1174-901-12	N/A	NA	
Beijerinckiaceae	Bosea	A	Bergey's Manual	
Beijerinckiaceae	Methylobacterium-Methy	A	Bergey's Manual	Based on Methylobacterium
Beijerinckiaceae	Microvirga	A	Kanso & Patel 2003	
Beijerinckiaceae	Other	A	Bergey's Manual	Based on Beijerinckiaceae
Beijerinckiaceae	Other	A	Bergey's manual	
Beijerinckiaceae	Roseiarcus	N/A		
Bifidobacteriaceae	Bifidobacterium	A	Bergey's Manual	
Bradymonadaceae	Bradymonas	N/A		

Brevibacteriaceae	Brevibacterium	A	Bergey's Manual	
Brevibacteriaceae	Spelaeicoccus	N/A		
Bryobacteraceae	Bryobacter	A	Bergey's Manual	
Budviciaceae	Budvicia	A	Bergey's Manual	
Butyricicoccaceae	Butyricicoccus	AN	Trachsel et al., 2018	
Butyricicoccaceae	Other	N/A	NA	
Butyricicoccaceae	Other	N/A		
Butyricicoccaceae	UCG-008	N/A	NA	
Butyricicoccaceae	UCG-009	AN	Bergey's Manual	Based on Clostridia
Caldicoprobacteraceae	Caldicoprobacter	AN	Bergey's Manual	
Candidatus Hepatincola	Other	N/A	NA	
Candidatus Hepatincola	Other	N/A		
Carnobacteriaceae	Atopostipes	A	Bergey's Manual	
Carnobacteriaceae	Carnobacterium	A	Bergey's Manual	
Carnobacteriaceae	Granulicatella	A	Bergey's Manual	
Carnobacteriaceae	Marinilactibacillus	A	Bergey's Manual	
Catellicoccaceae	Catellicoccus	N/A		
Caulobacteraceae	Brevundimonas	A	Bergey's Manual	
Caulobacteraceae	Caulobacter	A	Bergey's Manual	
Caulobacteraceae	Phenylobacterium	A	Bergey's Manual	
Cellulomonadaceae	Cellulomonas	A	Bergey's Manual	
Cellulomonadaceae	Oerskovia	A	Bergey's Manual	
Cellulomonadaceae	Paraoerskovia	A	Bergey's Manual	
Cellulomonadaceae	Pseudactinotalea	N/A		
Cellvibrionaceae	Cellvibrio	A	Bergey's Manual	
Chitinophagaceae	Chitinophaga	A	Bergey's Manual	Based on Chitinophagaceae
Chitinophagaceae	Haoranjiania	A	Bergey's Manual	
Chitinophagaceae	Other	A	Bergey's Manual	
Chitinophagaceae	Other	A	Bergey's manual	
Chitinophagaceae	Segetibacter	A	Bergey's Manual	
Christensenellaceae	Christensenella	AN	Morotomi et al., 2012	
Christensenellaceae	Christensenellaceae R-7 g	AN	Morotomi et al., 2012	
Christensenellaceae	Other	AN	Morotomi et al., 2012	
Christensenellaceae	Other	N/A		
Chroococcidiopsaceae	Aliterella	N/A		
Chthoniobacteraceae	Candidatus Udaeobacter	A	Bergey's Manual	
Clostridiaceae	Candidatus Arthromitus	AN	Schnupf et al., 2015	
Clostridiaceae	Clostridium sensu stricto	AN	Bergey's Manual	Based on Clostridiaceae
Clostridiaceae	Clostridium sensu stricto	AN	Bergey's Manual	Based on Clostridiaceae
Clostridiaceae	Clostridium sensu stricto	AN	Bergey's Manual	Based on Clostridiaceae
Clostridiaceae	Clostridium sensu stricto	AN	Bergey's Manual	Based on Clostridiaceae

Clostridiaceae	Other	AN	Bergey's Manual	Based on Clostridiaceae
Clostridiaceae	Other	AN	Bergey's manual	
Comamonadaceae	Comamonas	A	Bergey's Manual	
Comamonadaceae	Lampropedia	A	Bergey's Manual	
Comamonadaceae	Limnohabitans	A	Bergey's Manual	
Comamonadaceae	Other	N/A	Bergey's Manual	Based on Comamonadaceae
Comamonadaceae	Other	N/A		
Comamonadaceae	Polaromonas	A	Bergey's Manual	
Comamonadaceae	Simplicispira	N/A	Bergey's Manual	Based on Comamonadaceae
Comamonadaceae	Variovorax	A	Bergey's Manual	
Comamonadaceae	Xenophilus	N/A	Bergey's Manual	Based on Comamonadaceae
Coralloluteibacterium	Other	N/A	NA	
Coriobacteriaceae	Collinsella	AN	Bergey's Manual	
Coriobacteriales Incertae Sedis	Other	N/A	NA	
Corynebacteriaceae	Corynebacterium	A	Bergey's Manual	
Corynebacteriales Incertae Sedis	Tomitella	N/A		
Crocinitomicaceae	Fluviicola	A	Bergey's Manual	
Crocinitomicaceae	Other	N/A	NA	
Crocinitomicaceae	Other	N/A		
Cryomorphaceae	Other	A	Bergey's Manual	Based on Cryomorphaceae
Cryomorphaceae	Other	A	Bergey's manual	
Cyanobiaceae	Synechococcus CC9902	N/A		
Cyclobacteriaceae	Algoriphagus	A	Bergey's Manual	
Cyclobacteriaceae	Other	A	Bergey's Manual	Based on Cyclobacteriaceae
Cyclobacteriaceae	Other	A	Bergey's manual	
Deferribacteraceae	Mucispirillum	A	Bergey's Manual	Based on Deferribacteraceae
Defluviitaleaceae	Defluviitaleaceae UCG-01	A	Bergey's Manual	Based on Defluviitaleaceae
Demequinaceae	Demequina	N/A		
Dermabacteraceae	Brachybacterium	A	Bergey's Manual	
Dermabacteraceae	Helcobacillus	N/A	Bergey's Manual	Based on Dermabacteraceae
Dermacoccaceae	Flexivirga	A	Bergey's Manual	Based on Dermacoccaceae
Dermacoccaceae	Other	A	Bergey's Manual	Based on Dermacoccaceae
Desulfovibrionaceae	Bilophila	AN	Bergey's Manual	
Desulfovibrionaceae	Desulfovibrio	AN	Bergey's Manual	
Desulfovibrionaceae	Lawsonia	AN	Bergey's Manual	Based on Desulfovibrionaceae
Desulfovibrionaceae	Other	AN	Bergey's Manual	Based on Desulfovibrionaceae
DEV007	Other	N/A	NA	
Devosiaceae	Arsenicitalea	N/A		
Devosiaceae	Devosia	A	Bergey's Manual	
Devosiaceae	Other	N/A	NA	
Devosiaceae	Other	N/A		

Devosiaceae	Pelagibacterium	N/A		
Dietziaceae	Dietzia	A	Bergey's Manual	
Diplorickettsiaceae	Diplorickettsia	A	Mediannikov et al., 2010	
Diplorickettsiaceae	Other	N/A	NA	
Diplorickettsiaceae	Other	N/A		
Diplorickettsiaceae	Rickettsiella	A	Bergey's Manual	
Dysgonomonadaceae	Dysgonomonas	A	Bergey's Manual	
Eggerthellaceae	Adlercreutzia	AN	Bergey's Manual	
Eggerthellaceae	Asaccharobacter	A	Bergey's Manual	Based on Eggerthellaceae
Eggerthellaceae	DNF00809	N/A	Bergey's Manual	Based on Eggerthellaceae
Eggerthellaceae	Eggerthella	AN	Bergey's Manual	
Eggerthellaceae	Enterorhabdus	A	Clavel et al., 2009	
Eggerthellaceae	Gordonibacter	AN	Wurdemann et al., 2009, Bergey's Manual	
Eggerthellaceae	Other	A	Bergey's Manual	Based on Eggerthellaceae
Eggerthellaceae	Parvibacter	A	Clavel et al., 2013	
Enterobacteriaceae	Aquamonas	A	Bergey's Manual, Degelmann et a	Based on Enterobacteriaceae
Enterobacteriaceae	Atlantibacter	A	Bergey's Manual, Degelmann et a	Based on Enterobacteriaceae
Enterobacteriaceae	Buttiauxella	A	Bergey's Manual, Degelmann et al., 2009	
Enterobacteriaceae	Cedecea	A	Bergey's Manual, Degelmann et al., 2010	
Enterobacteriaceae	Citrobacter	A	Bergey's Manual, Degelmann et al., 2011	
Enterobacteriaceae	Escherichia-Shigella	A	Bergey's Manual, Degelmann et a	Based on Escherichia
Enterobacteriaceae	Klebsiella	A	Bergey's Manual, Degelmann et al., 2013	
Enterobacteriaceae	Kluyvera	A	Bergey's Manual, Degelmann et al., 2014	
Enterobacteriaceae	Kosakonia	A	Bergey's Manual, Degelmann et a	Based on Enterobacteriaceae
Enterobacteriaceae	Other	A	Bergey's Manual, Degelmann et a	Based on Enterobacteriaceae
Enterobacteriaceae	Other	A	Bergey's Manual, Degelmann et al., 2009	
Enterobacteriaceae	Raoultella	A	Bergey's Manual, Degelmann et a	Based on Enterobacteriaceae
Enterobacteriaceae	Salmonella	A	Bergey's Manual, Degelmann et al., 2015	
Enterobacteriaceae	Yokenella	A	Bergey's Manual, Degelmann et al., 2016	
Enterococcaceae	Enterococcus	A	Bergey's Manual	
Enterococcaceae	Other	N/A	NA	
Enterococcaceae	Other	N/A	Bergey's manual	N/A aerotolerance
Entomoplasmatales Incertae Sedis	Candidatus Hepatoplasm	N/A		
Erwiniaceae	Erwinia	A	Bergey's Manual	
Erwiniaceae	Other	N/A	NA	
Erwiniaceae	Other	N/A		
Erwiniaceae	Pantoea	A	Bergey's Manual	
Erwiniaceae	Siccibacter	N/A		
Erwiniaceae	Tatumella	A	Bergey's Manual	
Erysipelatoclostridiaceae	Candidatus Stoquefichus	A	Bergey's Manual	Based on Erysipelatoclostridiaceae
Erysipelatoclostridiaceae	Catenibacterium	AN	Bergey's Manual	

Erysipelatoclostridiaceae	Coprobacillus	AN	Bergey's Manual	
Erysipelatoclostridiaceae	Erysipelatoclostridium	AN	Yutin & Galperin, 2013	
Erysipelatoclostridiaceae	Erysipelotrichaceae UCG-	A	Bergey's Manual	Based on Erysipelatoclostridiaceae
Erysipelatoclostridiaceae	Other	A	Bergey's Manual	Based on Erysipelatoclostridiaceae
Erysipelatoclostridiaceae	Other	N/A		
Erysipelotrichaceae	[Clostridium] innocuum g	A	Bergey's Manual	Based on Erysipelotrichaceae
Erysipelotrichaceae	Dubosiella	AN	Cox et al., 2017	
Erysipelotrichaceae	Erysipelotrichaceae UCG-	A	Bergey's Manual	Based on Erysipelotrichaceae
Erysipelotrichaceae	Faecalibaculum	AN	Chang et al., 2015	
Erysipelotrichaceae	Faecalitalea	A	Bergey's Manual	Based on Erysipelotrichaceae
Erysipelotrichaceae	Holdemanella	A	Bergey's Manual	Based on Erysipelotrichaceae
Erysipelotrichaceae	Ileibacterium	A	Bergey's Manual	Based on Erysipelotrichaceae
Erysipelotrichaceae	Other	A	Bergey's Manual	Based on Erysipelotrichaceae
Erysipelotrichaceae	Other	A	Bergey's manual	
Erysipelotrichaceae	Turicibacter	A	Bergey's Manual	
Erysipelotrichaceae	ZOR0006	A	Bergey's Manual	Based on Erysipelotrichaceae
Eubacteriaceae	Other	AN	Bergey's Manual	Based on Eubacteriaceae
Eubacteriaceae	Other	AN	Bergey's manual	
Euzebyaceae	Other	N/A	NA	
Exiguobacteraceae	Exiguobacterium	A	Bergey's Manual	
Family XI	Other	N/A	NA	
Family XI	Tissierella	AN	Bergey's Manual	
Flavobacteriaceae	Aequorivita	A	Bergey's Manual	
Flavobacteriaceae	Aquibacter	A	Bergey's Manual	Based on Flavobacteriaceae
Flavobacteriaceae	Arenibacter	A	Bergey's Manual	
Flavobacteriaceae	Aurantiacicella	A	Bergey's Manual	Based on Flavobacteriaceae
Flavobacteriaceae	Flavobacterium	A	Bergey's Manual	
Flavobacteriaceae	Gelidibacter	A	Bergey's Manual	
Flavobacteriaceae	Gillisia	A	Bergey's Manual	
Flavobacteriaceae	Imtechella	A	Bergey's Manual	Based on Flavobacteriaceae
Flavobacteriaceae	Leeuwenhoekiella	A	Bergey's Manual	
Flavobacteriaceae	Muricauda	A	Bergey's Manual	
Flavobacteriaceae	Myroides	A	Bergey's Manual	
Flavobacteriaceae	Other	A	Bergey's Manual	Based on Flavobacteriaceae
Flavobacteriaceae	Other	N/A	Bergey's manual	
Flavobacteriaceae	Subsaxibacter	A	Bergey's Manual	
Frankiaceae	Frankia	A	Bergey's Manual	
Frankiaceae	Jatrophihabitans	A	Bergey's Manual	
Fusobacteriaceae	Cetobacterium	A	Bergey's Manual	
Fusobacteriaceae	Fusobacterium	AN	Bergey's Manual	
Gaiellaceae	Gaiella	A	Bergey's Manual	

Garciellaceae	Rhabdanaerobium	AN	Bergey's Manual	
Gemellaceae	Gemella	A	Bergey's Manual	
Geminicoccaceae	Candidatus Alysiosphaera	N/A		
Geminicoccaceae	Geminicoccus	N/A		
Geminicoccaceae	Other	N/A	NA	
Geminicoccaceae	Other	N/A		
Gemmataceae	Fimbriiglobus	A	Bergey's Manual	
Gemmataceae	Gemmata	A	Bergey's Manual	
Gemmataceae	Other	A	Bergey's Manual	Based on Gemmataceae
Gemmatimonadaceae	Gemmatimonas	A	Bergey's Manual	
Gemmatimonadaceae	Other	N/A	NA	
Geodermatophilaceae	Antricoccus	A	Bergey's Manual	Based on Geodermatophilaceae
Geodermatophilaceae	Blastococcus	A	Bergey's Manual	
Geodermatophilaceae	Klenkia	A	Bergey's Manual	
Geodermatophilaceae	Modestobacter	A	Bergey's Manual	
Gottschalkia	Other	N/A	NA	
Gottschalkia	Other	N/A		
Granulosicoccaceae	Granulosicoccus	N/A		
Hafniaceae	Edwardsiella	A	Bergey's Manual	
Hafniaceae	Hafnia-Obesumbacterium	A	Bergey's Manual	Based on Hafniaceae
Halomonadaceae	Chromohalobacter	A	Bergey's Manual	
Halomonadaceae	Halomonas	A	Bergey's Manual	
Halomonadaceae	Salinicola	A	Bergey's Manual	
Helicobacteraceae	Helicobacter	A	Bergey's Manual	
Hydrogenoanaerobacterium	Other	AN	Bergey's manual	
Hydrogenoanaerobacterium	Other	N/A	NA	
Hyphomicrobiaceae	Hyphomicrobium	A	Bergey's Manual	
Hyphomicrobiaceae	Pedomicrobium	A	Bergey's Manual	
Iamiaceae	Iamia	A	Bergey's Manual	
Ilumatobacteraceae	CL500-29 marine group	N/A		
Ilumatobacteraceae	Ilumatobacter	N/A		
Ilumatobacteraceae	Other	N/A	NA	
Intrasporangiaceae	Humibacillus	A	Bergey's Manual	
Intrasporangiaceae	Intrasporangium	A	Bergey's Manual	
Intrasporangiaceae	Janibacter	A	Bergey's Manual	
Intrasporangiaceae	Knoellia	A	Bergey's Manual	
Intrasporangiaceae	Oryzihumus	A	Bergey's Manual	
Intrasporangiaceae	Other	A	Bergey's Manual	Based on Intrasporangiaceae
Intrasporangiaceae	Pedococcus-Phycicoccus	A	Bergey's Manual	Based on Intrasporangiaceae
Isosphaeraceae	Aquisphaera	A	Bergey's Manual	
Isosphaeraceae	Candidatus Nostocoida	A	Bergey's Manual	Based on Isosphaeraceae

Isosphaeraceae	Isosphaera	A	Bergey's Manual	
Isosphaeraceae	Other	A	Bergey's Manual	Based on Isosphaeraceae
Isosphaeraceae	Paludisphaera	A	Bergey's Manual	
Isosphaeraceae	Singulisphaera	A	Bergey's Manual	
Isosphaeraceae	Tundrisphaera	A	Bergey's Manual	Based on Isosphaeraceae
JG30-KF-CM45	Other	N/A	NA	
Kaistiaceae	Kaistia	N/A		
Kineosporiaceae	Angustibacter	A	Bergey's Manual	
Kineosporiaceae	Other	N/A	NA	
Kineosporiaceae	Quadrisphaera	N/A	Bergey's Manual	
Ktedonobacteraceae	G12-WMSP1	A	Bergey's Manual	Based on Ktedonobacteraceae
Ktedonobacteraceae	HSB OF53-F07	A	Bergey's Manual	Based on Ktedonobacteraceae
Labraceae	Labrys	A	Bergey's Manual	
Lachnospiraceae	28-Apr	AN	Bergey's Manual	Based on Lachnospiraceae
Lachnospiraceae	[Acetivibrio] ethanolgigne	AN	Bergey's Manual	Based on Lachnospiraceae
Lachnospiraceae	[Eubacterium] eligens gr	AN	Bergey's Manual	Based on Lachnospiraceae
Lachnospiraceae	[Eubacterium] fissicatena	AN	Bergey's Manual	Based on Lachnospiraceae
Lachnospiraceae	[Eubacterium] hallii grou	AN	Bergey's Manual	Based on Lachnospiraceae
Lachnospiraceae	[Eubacterium] oxidoreduc	AN	Bergey's Manual	Based on Lachnospiraceae
Lachnospiraceae	[Eubacterium] ventriosun	AN	Bergey's Manual	Based on Lachnospiraceae
Lachnospiraceae	[Eubacterium] xylanophil	AN	Bergey's Manual	Based on Lachnospiraceae
Lachnospiraceae	[Ruminococcus] gauvreau	AN	Bergey's Manual	Based on Lachnospiraceae
Lachnospiraceae	[Ruminococcus] gnavus g	AN	Bergey's Manual	Based on Lachnospiraceae
Lachnospiraceae	[Ruminococcus] torques g	AN	Bergey's Manual	Based on Lachnospiraceae
Lachnospiraceae	A2	AN	Bergey's Manual	Based on Lachnospiraceae
Lachnospiraceae	Acetatifactor	AN	Bergey's Manual	Based on Lachnospiraceae
Lachnospiraceae	Agathobacter	AN	Bergey's Manual	Based on Lachnospiraceae
Lachnospiraceae	Anaerosporobacter	AN	Bergey's Manual	Based on Lachnospiraceae
Lachnospiraceae	Anaerostipes	AN	Bergey's Manual	Based on Lachnospiraceae
Lachnospiraceae	ASF356	AN	Bergey's Manual	Based on Lachnospiraceae
Lachnospiraceae	Blautia	AN	Bergey's Manual	Based on Lachnospiraceae
Lachnospiraceae	Cellulosilyticum	AN	Bergey's Manual	Based on Lachnospiraceae
Lachnospiraceae	Coprococcus	AN	Bergey's Manual	Based on Lachnospiraceae
Lachnospiraceae	Cuneatibacter	AN	Bergey's Manual	Based on Lachnospiraceae
Lachnospiraceae	Dorea	AN	Bergey's Manual	Based on Lachnospiraceae
Lachnospiraceae	Eisenbergiella	AN	Bergey's Manual	Based on Lachnospiraceae
Lachnospiraceae	Epulopiscium	AN	Bergey's Manual	Based on Lachnospiraceae
Lachnospiraceae	Fusicatenibacter	AN	Bergey's Manual	Based on Lachnospiraceae
Lachnospiraceae	GCA-900066575	AN	Bergey's Manual	Based on Lachnospiraceae
Lachnospiraceae	Hungatella	AN	Bergey's Manual	Based on Lachnospiraceae
Lachnospiraceae	Lachnoclostridium	AN	Bergey's Manual	Based on Lachnospiraceae

Lachnospiraceae	Lachnospiraceae FCS020	AN	Bergey's Manual	Based on Lachnospiraceae
Lachnospiraceae	Lachnospiraceae NK4A13	AN	Bergey's Manual	Based on Lachnospiraceae
Lachnospiraceae	Lachnospiraceae NK4B4 g	AN	Bergey's Manual	Based on Lachnospiraceae
Lachnospiraceae	Lachnospiraceae UCG-00	AN	Bergey's Manual	Based on Lachnospiraceae
Lachnospiraceae	Lachnospiraceae UCG-00	AN	Bergey's Manual	Based on Lachnospiraceae
Lachnospiraceae	Lachnospiraceae UCG-00	AN	Bergey's Manual	Based on Lachnospiraceae
Lachnospiraceae	Lachnospiraceae UCG-00	AN	Bergey's Manual	Based on Lachnospiraceae
Lachnospiraceae	Lachnospiraceae UCG-00	AN	Bergey's Manual	Based on Lachnospiraceae
Lachnospiraceae	Lachnospiraceae UCG-01	AN	Bergey's Manual	Based on Lachnospiraceae
Lachnospiraceae	Marvinbryantia	AN	Bergey's Manual	Based on Lachnospiraceae
Lachnospiraceae	Murimonas	AN	Bergey's Manual	Based on Lachnospiraceae
Lachnospiraceae	Other	AN	Bergey's Manual	Based on Lachnospiraceae
Lachnospiraceae	Other	AN	Bergey's manual	
Lachnospiraceae	possible genus Sk018	AN	Bergey's Manual	Based on Lachnospiraceae
Lachnospiraceae	Robinsoniella	AN	Bergey's Manual	Based on Lachnospiraceae
Lachnospiraceae	Roseburia	AN	Bergey's Manual	Based on Lachnospiraceae
Lachnospiraceae	Sellimonas	AN	Bergey's Manual	Based on Lachnospiraceae
Lachnospiraceae	Tuzzerella	AN	Bergey's Manual	Based on Lachnospiraceae
Lachnospiraceae	Tyzzerella	AN	Bergey's Manual	Based on Lachnospiraceae
Lactobacillaceae	Agrilactobacillus	N/A	Bergey's Manual	Based on Lactobacillaceae
Lactobacillaceae	Bombilactobacillus	N/A	Bergey's Manual	Based on Lactobacillaceae
Lactobacillaceae	Companilactobacillus	N/A	Bergey's Manual	Based on Lactobacillaceae
Lactobacillaceae	Dellaglioa	N/A	Bergey's Manual	Based on Lactobacillaceae
Lactobacillaceae	HT002	N/A	Bergey's Manual	Based on Lactobacillaceae
Lactobacillaceae	Lacticaseibacillus	N/A	Bergey's Manual	Based on Lactobacillaceae
Lactobacillaceae	Lactiplantibacillus	N/A	Bergey's Manual	Based on Lactobacillaceae
Lactobacillaceae	Lactobacillus	A	Bergey's Manual	
Lactobacillaceae	Latilactobacillus	N/A	Bergey's Manual	Based on Lactobacillaceae
Lactobacillaceae	Leuconostoc	N/A	Bergey's Manual	
Lactobacillaceae	Levilactobacillus	N/A	Bergey's Manual	Based on Lactobacillaceae
Lactobacillaceae	Ligilactobacillus	A	Aerotolerance N/A based on Marta et al., 2021; Zheng et al., 2020 - so	
Lactobacillaceae	Limosilactobacillus	A	aerotolerance was N/A (Zheng et a., 2020) so BLASTed and blasts agai	
Lactobacillaceae	Other	N/A	Bergey's Manual	Based on Lactobacillaceae
Lactobacillaceae	Other	N/A		
Lactobacillaceae	Paucilactobacillus	N/A	Bergey's Manual	Based on Lactobacillaceae
Lactobacillaceae	Weissella	A	Bergey's Manual	
Legionellaceae	Legionella	A	Bergey's Manual	
Leptolyngbyaceae	Leptolyngbya PCC-6306	N/A		
Listeriaceae	Listeria	A	Bergey's Manual	Based on Listeriaceae
Marinifilaceae	Butyricimonas	N/A	NA	
Marinifilaceae	Odoribacter	AN	Hardham et al., 2008	

Marinifilaceae	Other	N/A	NA	
Marinifilaceae	Other	N/A		
Marinifilaceae	Sanguibacteroides	N/A		
Marinilabiliaceae	Natronoflexus	N/A	Bergey's Manual	Based on Marinilabiliaceae
Marinilabiliaceae	Other	N/A	Bergey's Manual	Based on Marinilabiliaceae
Marinilabiliaceae	Other	N/A		
Marinobacteraceae	Marinobacter	A	Bergey's Manual	
Methyloligellaceae	Methyloligella	N/A		
Methyloligellaceae	Other	N/A	NA	
Methyloligellaceae	Other	N/A		
Microbacteriaceae	Agrococcus	A	Bergey's Manual	
Microbacteriaceae	Amnibacterium	A	Bergey's Manual	
Microbacteriaceae	Curtobacterium	A	Bergey's Manual	
Microbacteriaceae	Frigoribacterium	A	Bergey's Manual	
Microbacteriaceae	Homoserinibacter	A	Bergey's Manual	
Microbacteriaceae	Leucobacter	N/A	Bergey's Manual	
Microbacteriaceae	Lysinimonas	A	Bergey's Manual	
Microbacteriaceae	Microbacterium	A	Bergey's Manual	
Microbacteriaceae	Microterricola	A	Bergey's Manual	
Microbacteriaceae	Mycetocola	A	Bergey's Manual	
Microbacteriaceae	Other	A	Bergey's Manual	Based on Microbacteriaceae
Microbacteriaceae	Parafrigoribacterium	A	Bergey's Manual	Based on Microbacteriaceae
Microbacteriaceae	Plantibacter	A	Bergey's Manual	
Microbacteriaceae	Pseudoclavibacter	A	Bergey's Manual	
Micrococcaceae	Arthrobacter	A	Bergey's Manual	
Micrococcaceae	Glutamicibacter	A	Bergey's Manual	
Micrococcaceae	Kocuria	A	Bergey's Manual	
Micrococcaceae	Nesterenkonia	A	Bergey's Manual	
Micrococcaceae	Other	N/A	NA	
Micrococcaceae	Paeniglutamicibacter	A	Bergey's Manual	
Micrococcaceae	Pseudarthrobacter	A	Bergey's Manual	
Micromonosporaceae	Actinoplanes	A	Bergey's Manual	
Micromonosporaceae	Micromonospora	A	Bergey's Manual	
Micromonosporaceae	Other	A	Bergey's Manual	Based on Micromonosporaceae
Micromonosporaceae	Xiangella	A	Bergey's Manual	
Monoglobaceae	Monoglobus	AN	Kim et al., 2017	
Moraxellaceae	Acinetobacter	A	Bergey's Manual	
Moraxellaceae	Alkanindiges	A	Bergey's Manual	Based on Moraxellaceae
Moraxellaceae	Enhydrobacter	A	Bergey's Manual	
Moraxellaceae	Psychrobacter	A	Bergey's Manual	
Morganellaceae	Cosenzaea	N/A		

Morganellaceae	Moellerella	A	Bergey's Manual	
Morganellaceae	Morganella	A	Bergey's Manual	
Morganellaceae	Other	N/A	NA	
Morganellaceae	Other	N/A		
Morganellaceae	Proteus	A	Bergey's Manual	
Morganellaceae	Providencia	A	Bergey's Manual	
Muribaculaceae	Muribaculum	AN	Bergey's Manual	
Muribaculaceae	Other	AN	Lagkouvardos et al 2019	
Muribaculaceae	Other	AN	Lagkouvardos et al., 2019	
MWH-CFBk5	Other	N/A	NA	
MWH-CFBk5	Other	N/A		
Mycobacteriaceae	Mycobacterium	A	Bergey's Manual	
Mycobacteriaceae	Other	N/A		
Mycoplasmataceae	Candidatus Bacilloplasma	A	Bergey's Manual	Based on Mycoplasmataceae
Mycoplasmataceae	Mycoplasma	N/A	Bergey's Manual	
Mycoplasmataceae	Other	A	Bergey's Manual	Based on Mycoplasmataceae
Mycoplasmataceae	Other	A	Bergey's manual	
Myxococcaceae	P3OB-42	N/A		
Nakamurellaceae	Nakamurella	A	Bergey's Manual	
Nannocystaceae	Enhygromyxa	N/A		
Neisseriaceae	Other	A	Bergey's Manual	Based on Neisseriaceae
Neisseriaceae	Other	A	Bergey's manual	
Neisseriaceae	Vitreoscilla	A	Bergey's Manual	
Nitrospiraceae	Nitrospira	A	Bergey's Manual	
Nocardiaceae	Gordonia	A	Bergey's Manual	
Nocardiaceae	Nocardia	A	Bergey's Manual	
Nocardiaceae	Other	A	Bergey's Manual	Based on Nocardiaceae
Nocardiaceae	Rhodococcus	A	Bergey's Manual	
Nocardiaceae	Williamsia	A	Bergey's Manual	
Nocardioidaceae	Aeromicrobium	A	Bergey's Manual	
Nocardioidaceae	Marmoricola	A	Bergey's Manual	
Nocardioidaceae	Mumia	A	Bergey's Manual	Based on Nocardioidaceae
Nocardioidaceae	Nocardioides	A	Bergey's Manual	
Nocardioidaceae	Other	A	Bergey's Manual	Based on Nocardioidaceae
Nocardiopsaceae	Nocardiopsis	A	Bergey's Manual	
Nostocaceae	Calothrix PCC-6303	N/A		
Nostocaceae	Other	N/A	NA	
Nostocaceae	Rivularia PCC-7116	N/A	Bergey's Manual	Based on Rivularia
Oligoflexaceae	Oligoflexus	N/A		
Oscillospiraceae	Colidextribacter	AN	Ricaboni et al., 2017	
Oscillospiraceae	Flavonifractor	AN	Bergey's Manual	Based on Eubacterium

Oscillospiraceae	Intestinimonas	AN	Kläring et al., 2013, Bergey's manual	
Oscillospiraceae	NK4A214 group	AN	Tindal et al., 2019	
Oscillospiraceae	Oscillibacter	AN	Iino et al. 2007	
Oscillospiraceae	Oscillospira	AN	Bergey's Manual	
Oscillospiraceae	Other	A	Bergey's Manual	Based on Oscillospiraceae
Oscillospiraceae	Other	N/A		
Oscillospiraceae	Papillibacter	AN	Bergey's Manual	
Oscillospiraceae	Pseudoflavonifractor	N/A		
Oscillospiraceae	UCG-002	N/A	NA	
Oscillospiraceae	UCG-003	N/A	NA	
Oscillospiraceae	UCG-005	AN	Bergey's Manual	Based on Oscillospiraceae
Oscillospiraceae	UCG-007	N/A	NA	
Other	Other	N/A	NA	
Oxalobacteraceae	Duganella	A	Bergey's Manual	
Oxalobacteraceae	Massilia	A	Bergey's Manual	
Oxalobacteraceae	Noviherbaspirillum	A	Bergey's Manual	Based on Oxalobacteraceae
Oxalobacteraceae	Oxalicibacterium	A	Bergey's Manual	Based on Oxalobacteraceae
Oxalobacteraceae	Oxalobacter	AN	Bergey's Manual	
Oxalobacteraceae	Paraherbaspirillum	A	Bergey's Manual	Based on Oxalobacteraceae
Paenibacillaceae	Ammoniphilus	A	Bergey's Manual	
Paenibacillaceae	Paenibacillus	A	Bergey's Manual	
Paludibacteraceae	H1	N/A		
Pasteurellaceae	Conservatibacter	N/A	Bergey's Manual	
Pasteurellaceae	Mesocricetibacter	A	Bergey's Manual	
Pasteurellaceae	Muribacter	N/A	Bergey's Manual	
Pasteurellaceae	Rodentibacter	A	Bergey's Manual, Benga, Sager & Christensen 2018	
Pectobacteriaceae	Nissabacter	N/A		
Peptococcaceae	Other	AN	Bergey's Manual	Based on Peptococcaceae
Peptococcaceae	Other	AN	Bergey's manual	
Peptococcaceae	Peptococcus	AN	Bergey's Manual	
Peptostreptococcaceae	[Eubacterium] tenue grou	N/A	NA	
Peptostreptococcaceae	Clostridioides	A	Bergey's Manual	Based on Peptostreptococcaceae
Peptostreptococcaceae	Intestinibacter	A	Bergey's Manual	Based on Peptostreptococcaceae
Peptostreptococcaceae	Paeniclostridium	A	Bergey's Manual	Based on Peptostreptococcaceae
Peptostreptococcaceae	Paraclostridium	A	Bergey's Manual	
Peptostreptococcaceae	Romboutsia	AN	Gerritsen et al., BioRxiv 2019	
Peptostreptococcaceae	Sporacetigenium	AN	Bergey's Manual	
Peptostreptococcaceae	Terrisporobacter	A	Bergey's Manual	Based on Peptostreptococcaceae
Phormidesmiaceae	Phormidesmis ANT.LACV	N/A		
Phormidiaceae	Other	N/A	NA	
Phormidiaceae	Tychonema CCAP 1459-1	N/A	NA	

Pirellulaceae	Blastopirellula	A	Bergey's Manual	
Pirellulaceae	Bythopirellula	N/A	NA	
Pirellulaceae	Other	N/A	NA	
Pirellulaceae	Pir4 lineage	N/A	Bergey's Manual	Based on Pirellulaceae
Pirellulaceae	Pirellula	A	Bergey's Manual	
Pirellulaceae	Rhodopirellula	A	Bergey's Manual	
Pirellulaceae	Rubripirellula	A	Bergey's Manual	
Planococcaceae	Kurthia	A	Bergey's Manual	
Planococcaceae	Lysinibacillus	A	Bergey's Manual	
Planococcaceae	Other	A	Bergey's manual	
Planococcaceae	Other	N/A		
Planococcaceae	Paenisporosarcina	A	Krishnamurthi et al., 2009	
Planococcaceae	Planomicrobium	A	Bergey's Manual	
Planococcaceae	Psychrobacillus	A	Bergey's Manual	Based on Planococcaceae
Planococcaceae	Solibacillus	A	Krishnamurthi et al., 2009	
Planococcaceae	Sporosarcina	A	Bergey's Manual	
Polyangiaceae	Aetherobacter	A	Garcia et al.	
Porphyromonadaceae	Falsiporphyromonas	AN	Bergey's Manual	Based on Porphyromonadaceae
Porphyromonadaceae	Other	AN	Bergey's Manual	Based on Porphyromonadaceae
Porphyromonadaceae	Other	AN	Bergey's manual	
Prevotellaceae	Alloprevotella	AN	Bergey's Manual	Based on Prevotellaceae
Prevotellaceae	Other	A	Bergey's Manual	Based on Prevotellaceae
Prevotellaceae	Other	AN	Bergey's manual	
Prevotellaceae	Paraprevotella	AN	Bergey's Manual	Based on Prevotellaceae
Prevotellaceae	Prevotella_7	A	Bergey's Manual	Based on Prevotella
Prevotellaceae	Prevotella_9	A	NA	
Prevotellaceae	Prevotellaceae UCG-001	A	Bergey's Manual	Based on Prevotellaceae
Prevotellaceae	Prevotellaceae UCG-004	A	Bergey's Manual	Based on Prevotellaceae
Promicromonosporaceae	Isoptericola	A	Bergey's Manual	
Propionibacteriaceae	Friedmanniella	A	Bergey's Manual	
Propionibacteriaceae	Marinilutecoccus	N/A		
Propionibacteriaceae	Microlunatus	A	Bergey's Manual	
Propionibacteriaceae	Other	N/A	NA	
Propionibacteriaceae	Tessaracoccus	A	Bergey's Manual	
Pseudomonadaceae	Pseudomonas	A	Bergey's Manual	
Pseudonocardiaceae	Actinomycetospora	A	Bergey's Manual	
Pseudonocardiaceae	Actinophytocola	A	Bergey's Manual	Based on Pseudonocardiaceae
Pseudonocardiaceae	Crossiella	A	Bergey's Manual	Based on Pseudonocardiaceae
Pseudonocardiaceae	Kibdelosporangium	A	Bergey's Manual	
Pseudonocardiaceae	Other	A	Bergey's Manual	Based on Pseudonocardiaceae
Pseudonocardiaceae	Pseudonocardia	A	Bergey's Manual	

Puniceicoccaceae	Cerasicoccus	A	Bergey's Manual	
Reyranellaceae	Reyranella	N/A		
Rhizobiaceae	Ahrensia	A	Bergey's Manual	
Rhizobiaceae	Aliihoeflea	A	Bergey's Manual	Based on Rhizobiaceae
Rhizobiaceae	Allorhizobium-Neorhizob	A	Bergey's Manual	Based on Rhizobium and Allorhizobi
Rhizobiaceae	Aminobacter	A	Bergey's Manual	
Rhizobiaceae	Aquamicrobium	N/A	Bergey's Manual	
Rhizobiaceae	Aurantimonas	A	Bergey's Manual	Based on Rhizobiaceae
Rhizobiaceae	Aureimonas	A	Bergey's Manual	Based on Rhizobiaceae
Rhizobiaceae	Brucella	A	Bergey's Manual	
Rhizobiaceae	Corticibacterium	A	Bergey's Manual	Based on Rhizobiaceae
Rhizobiaceae	Falsochrobactrum	N/A	Bergey's Manual	
Rhizobiaceae	Hoeflea	A	Bergey's Manual	Based on Rhizobiaceae
Rhizobiaceae	Jiella	A	Bergey's Manual	Based on Rhizobiaceae
Rhizobiaceae	Mesorhizobium	A	Bergey's Manual	
Rhizobiaceae	Neorhizobium	A	Bergey's Manual	Based on Rhizobiaceae
Rhizobiaceae	Ochrobactrum	A	Bergey's Manual	
Rhizobiaceae	Other	A	Bergey's Manual	Based on Rhizobiaceae
Rhizobiaceae	Other	A	Bergey's manual	
Rhizobiaceae	Paenochrobactrum	A	Bergey's Manual	
Rhizobiaceae	Phyllobacterium	A	Bergey's Manual	
Rhizobiaceae	Pseudaminobacter	A	Bergey's Manual	
Rhizobiaceae	Shinella	A	Bergey's Manual	Based on Rhizobiaceae
Rhizobiaceae	Tianweitania	A	Bergey's Manual	Based on Rhizobiaceae
Rhodanobacteraceae	Chujaibacter	N/A		
Rhodanobacteraceae	Dokdonella	N/A		
Rhodanobacteraceae	Luteibacter	N/A		
Rhodanobacteraceae	Mizugakiibacter	N/A		
Rhodanobacteraceae	Oleiagrimonas	N/A		
Rhodanobacteraceae	Rhodanobacter	A	Bergey's Manual	
Rhodobacteraceae	Actibacterium	A	Bergey's Manual	Based on Rhodobacteraceae
Rhodobacteraceae	Albirhodobacter	A	Bergey's Manual	Based on Rhodobacteraceae
Rhodobacteraceae	Amaricoccus	A	Bergey's Manual	
Rhodobacteraceae	Boseongicola	A	Bergey's Manual	Based on Rhodobacteraceae
Rhodobacteraceae	Defluviimonas	A	Bergey's Manual	Based on Rhodobacteraceae
Rhodobacteraceae	Falsirhodobacter	A	Bergey's Manual	Based on Rhodobacteraceae
Rhodobacteraceae	Gemmobacter	A	Bergey's Manual	
Rhodobacteraceae	Jannaschia	A	Bergey's Manual	Based on Rhodobacteraceae
Rhodobacteraceae	Limibaculum	A	Bergey's Manual	Based on Rhodobacteraceae
Rhodobacteraceae	Maribius	A	Bergey's Manual	Based on Rhodobacteraceae
Rhodobacteraceae	Oceaniovalibus	A	Bergey's Manual	Based on Rhodobacteraceae

Rhodobacteraceae	Octadecabacter	A	Bergey's Manual	
Rhodobacteraceae	Other	A	Bergey's Manual	Based on Rhodobacteraceae
Rhodobacteraceae	Other	A	Bergey's manual	
Rhodobacteraceae	Paenirhodobacter	A	Bergey's Manual	Based on Rhodobacteraceae
Rhodobacteraceae	Paracoccus	A	Bergey's Manual	
Rhodobacteraceae	Plastorhodobacter	A	Bergey's Manual	Based on Rhodobacteraceae
Rhodobacteraceae	Pseudorhodobacter	A	Bergey's Manual	Based on Rhodobacteraceae
Rhodobacteraceae	Pseudoruegeria	A	Bergey's Manual	Based on Rhodobacteraceae
Rhodobacteraceae	Rhodobacter	N/A	Bergey's Manual	
Rhodobacteraceae	Rhodobaculum	A	Bergey's Manual	Based on Rhodobacteraceae
Rhodobacteraceae	Roseivivax	A	Bergey's Manual	
Rhodobacteraceae	Roseovarius	A	Bergey's Manual	
Rhodobacteraceae	Rubellimicrobium	A	Bergey's Manual	Based on Rhodobacteraceae
Rhodobacteraceae	Sulfitobacter	A	Bergey's Manual	
Rhodobacteraceae	Thioclava	A	Bergey's Manual	Based on Rhodobacteraceae
Rhodobacteraceae	Yoonia-Loktanella	A	Bergey's Manual	Based on Yoonia
Rhodocyclaceae	Azovibrio	A	Bergey's Manual	
Rhodocyclaceae	Thauera	A	Bergey's Manual	
Rhodomicrobiaceae	Rhodomicrobium	A	Bergey's Manual	
Rhodothermaceae	Rubrivirga	A	Bergey's Manual	Based on Rhodothermaceae
Rickettsiaceae	Rickettsia	N/A	Bergey's Manual	
Rikenellaceae	Alistipes	AN	Bergey's Manual	
Rikenellaceae	Other	N/A		
Rikenellaceae	Other	N/A		
Rikenellaceae	Rikenella	AN	Bergey's Manual	
Rikenellaceae	Rikenellaceae RC9 gut gr	A	Bergey's Manual	Based on Rikenellaceae
Rs-E47 termite group	Other	N/A	NA	
Rs-E47 termite group	Other	N/A		
Rubinisphaeraceae	Planctomicrobium	N/A		
Rubinisphaeraceae	SH-PL14	N/A		
Rubritaleaceae	Luteolibacter	A	Bergey's Manual	Based on Rubritaleaceae
Ruminococcaceae	[Eubacterium] siraeum g	AN	Bergey's Manual	Based on Eubacterium
Ruminococcaceae	Anaerotruncus	AN	Bergey's Manual	
Ruminococcaceae	Angelakisella	AN	Bergey's Manual	Based on Ruminococcaceae
Ruminococcaceae	Candidatus Soleaferrea	AN	Bergey's Manual	Based on Ruminococcaceae
Ruminococcaceae	Caproiciproducens	AN	Bergey's Manual	Based on Ruminococcaceae
Ruminococcaceae	DTU089	AN	Bergey's Manual	Based on Ruminococcaceae
Ruminococcaceae	Faecalibacterium	AN	Bergey's Manual	
Ruminococcaceae	Fournierella	AN	Bergey's Manual	Based on Ruminococcaceae
Ruminococcaceae	Harryflintia	AN	Bergey's Manual	Based on Ruminococcaceae
Ruminococcaceae	Incertae Sedis	AN	Bergey's Manual, Browne et al., 2(	Based on Ruminococcaceae

Ruminococcaceae	Negativibacillus	AN	Bergey's Manual	Based on Ruminococcaceae
Ruminococcaceae	Other	AN	Bergey's Manual	Based on Ruminococcaceae
Ruminococcaceae	Other	AN	Bergey's manual	
Ruminococcaceae	Paludicola	AN	Bergey's Manual	Based on Ruminococcaceae
Ruminococcaceae	Pygmaiobacter	AN	Bergey's Manual	Based on Ruminococcaceae
Ruminococcaceae	Ruminococcus	AN	Bergey's Manual, Browne et al., 2016	
Ruminococcaceae	Subdoligranulum	AN	Bergey's Manual	
Ruminococcaceae	UBA1819	AN	Bergey's Manual	Based on Faecalibacterium
Salinisphaeraceae	Salinisphaera	A	Bergey's Manual	
Sandaracinaceae	Other	N/A	NA	
Sanguibacteraceae	Sanguibacter-Flavimobili	N/A	Bergey's Manual	Based on Sanguibacter
Saprospiraceae	Lewinella	A	Bergey's Manual	Based on Saprospiraceae
Saprospiraceae	Other	A	Bergey's Manual	Based on Saprospiraceae
Saprospiraceae	Other	A	Bergey's manual	
SC-I-84	Other	N/A	NA	
SC-I-84	Other	N/A		
Schlesneriaceae	Planctopirus	N/A		
Schlesneriaceae	Schlesneria	A	Bergey's Manual	
Shewanellaceae	Shewanella	A	Bergey's Manual	
Solirubrobacteraceae	Conexibacter	A	Bergey's Manual	
Solirubrobacteraceae	Other	N/A	Bergey's Manual	Based on Solirubrobacteraceae
Solirubrobacteraceae	Parviterribacter	N/A	NA	
Solirubrobacteraceae	Patulibacter	A	Bergey's Manual	
Solirubrobacteraceae	Solirubrobacter	A	Bergey's Manual	
Sphingobacteriaceae	Pedobacter	A	Bergey's Manual	
Sphingobacteriaceae	Sphingobacterium	A	Bergey's Manual	
Sphingomonadaceae	Altererythrobacter	N/A		
Sphingomonadaceae	Erythrobacter	A	Bergey's Manual	
Sphingomonadaceae	Novosphingobium	A	Takeuchi et al., 2001, Bergey's manual	
Sphingomonadaceae	Qipengyuania	N/A		
Sphingomonadaceae	Sphingomonas	A	Bergey's Manual	
Sphingomonadaceae	Sphingopyxis	A	Bergey's Manual	
Spirosomaceae	Persicitalea	N/A		
Spirosomaceae	Rhabdobacter	N/A		
Sporichthyaceae	Longivirga	A	Bergey's Manual	Based on Sporichthyaceae
Sporichthyaceae	Other	A	Bergey's Manual	Based on Sporichthyaceae
Sporomusaceae	Dendrosporobacter	AN	Bergey's Manual	
Sporomusaceae	Other	N/A	NA	
Sporomusaceae	Other	N/A		
Sporomusaceae	Sporomusa	A	Bergey's Manual	
Staphylococcaceae	Corticicoccus	N/A		

Staphylococcaceae	Jeotgalicoccus	A	Bergey's Manual	
Staphylococcaceae	Macrococcus	N/A		
Staphylococcaceae	Other	N/A	NA	
Staphylococcaceae	Other	N/A		
Staphylococcaceae	Staphylococcus	A	Bergey's Manual	
Stappiaceae	Labrenzia	N/A		
Stappiaceae	Other	N/A	NA	
Streptococcaceae	Lactococcus	A	Bergey's Manual	
Streptococcaceae	Other	A	Bergey's Manual	Based on Streptococcaceae
Streptococcaceae	Other	A	Bergey's manual	
Streptococcaceae	Streptococcus	A	Bergey's Manual	
Streptomycetaceae	Kitasatospora	A	Bergey's Manual	Based on Streptomycetaceae
Streptomycetaceae	Other	A	Bergey's Manual	Based on Streptomycetaceae
Streptomycetaceae	Streptomyces	A	Bergey's Manual	
Streptosporangiaceae	Streptosporangium	A	Bergey's Manual	
Sulfobacillaceae	Other	N/A	NA	
Sulfobacillaceae	Other	N/A		
Sumerlaeaceae	Sumerlaea	N/A		
Sutterellaceae	Other	N/A	NA	
Sutterellaceae	Other	N/A		
Sutterellaceae	Parasutterella	A	Nagai et al., 2009	
Sutterellaceae	Sutterella	A	Bergey's Manual	
Tannerellaceae	Candidatus Vestibaculum	N/A		
Tannerellaceae	Macellibacteroides	N/A		
Tannerellaceae	Other	N/A	NA	
Tannerellaceae	Other	N/A		
Tannerellaceae	Parabacteroides	AN	Sakamoto & Benno, 2006	
Thermoactinomycetaceae	Other	A	Bergey's Manual	Based on Thermoactinomycetaceae
Thermoactinomycetaceae	Other	A	Bergey's manual	
Thermoactinomycetaceae	Risungbinella	A	Bergey's Manual	Based on Thermoactinomycetaceae
Trueperaceae	Truepera	A	Bergey's Manual	
Tsukamurellaceae	Tsukamurella	A	Bergey's Manual	
UCG-010	Other	N/A	NA	
UCG-010	Other	N/A		
Vagococcaceae	Vagococcus	A	Bergey's Manual	
Veillonellaceae	Dialister	AN	Bergey's Manual	
Veillonellaceae	Veillonella	A	Bergey's Manual	
Vibrionaceae	Vibrio	A	Bergey's Manual	
WD2101 soil group	Other	N/A	NA	
Weeksellaceae	Candidatus Hemobacteriu	N/A		
Weeksellaceae	Chishuiella	N/A		

Weeksellaceae	Chryseobacterium	A	Bergey's Manual	
Weeksellaceae	Empedobacter	A	Bergey's Manual	
Wohlfahrtiimonadaceae	Ignatzschineria	N/A		
Wohlfahrtiimonadaceae	Wohlfahrtiimonas	N/A		
Xanthobacteraceae	Afipia	A	NA	
Xanthobacteraceae	Bradyrhizobium	A	Bergey's Manual	
Xanthobacteraceae	Other	N/A	NA	
Xanthobacteraceae	Other	N/A		
Xanthobacteraceae	Pseudolabrys	N/A		
Xanthobacteraceae	Pseudorhodoplanes	N/A		
Xanthobacteraceae	Rhodoplanes	A	Bergey's Manual	
Xanthobacteraceae	Rhodopseudomonas	A	Bergey's Manual	
Xanthomonadaceae	Luteimonas	A	Bergey's Manual	
Xanthomonadaceae	Lysobacter	A	Bergey's Manual	
Xanthomonadaceae	Pseudoxanthomonas	A	Bergey's Manual	
Xanthomonadaceae	SN8	N/A		
Xanthomonadaceae	Stenotrophomonas	A	Bergey's Manual	
Xanthomonadaceae	Thermomonas	A	Bergey's Manual	
Xenococcaceae	Pleurocapsa PCC-7319	A	Bergey's Manual	
Yersiniaceae	Other	N/A	NA	
Yersiniaceae	Other	N/A		
Yersiniaceae	Rahnella	A	Bergey's Manual	
Yersiniaceae	Serratia	A	Bergey's Manual	
Yersiniaceae	Yersinia	A	Bergey's Manual	

References

Benga, L., Sager, M., & Christensen, H. (2018). From the [Pasteurella] pneumotropica complex to Rodentibacter spp.: an update on [Pasteurella] pneumotropica. In *Veterinary Microbiology* (Vol. 217, pp. 121–134). Elsevier B.V. https://doi.org/10.1016/j.vetmic.2018.03.011

Browne, H. P., Forster, S. C., Anonye, B. O., Kumar, N., Neville, B. A., Stares, M. D., Goulding, D., & Lawley, T. D. (2016). Culturing of "unculturable" human microbiota reveals novel taxa and extensive sporulation. *Nature*, *533*(7604), 543–546. https://doi.org/10.1038/nature17645

Cai, S., & Dong, X. (2010). Cellulosilyticum ruminicola gen. nov., sp. nov., isolated from the rumen of yak, and reclassification of Clostridium lentocellum as Cellulosilyticum lentocellum comb. nov. *International Journal of Systematic and Evolutionary Microbiology*, *60*(4), 845–849. https://doi.org/10.1099/ijs.0.014712-0

Chang DH, Rhee MS, Ahn S, et al. Faecalibaculum rodentium gen. nov., sp. nov., isolated from the faeces of a laboratory mouse [published correction appears in Antonie Van Leeuwenhoek. 2016 Mar;109(3):481]. *Antonie Van Leeuwenhoek*. 2015;108(6):1309-1318. doi:10.1007/s10482-015-0583-3

Clavel, T., Charrier, C., Wenning, M., & Haller, D. (2013). Parvibacter caecicola gen. nov., sp. nov., a bacterium of the family Coriobacteriaceae isolated from the caecum of a mouse. *International Journal of Systematic and Evolutionary Microbiology*, *63*(PART7), 2642–2648. https://doi.org/10.1099/ijs.0.045344-0

Clavel, T., Duck, W., Charrier, C., Wenning, M., Elson, C., & Haller, D. (2010). Enterorhabdus caecimuris sp. nov., a member of the family Coriobacteriaceae isolated from a mouse model of spontaneous colitis, and emended description of the genus Enterorhabdus Clavel et al. 2009. In *International Journal of Systematic and Evolutionary Microbiology* (Vol. 60, Issue 7, pp. 1527– 1531). Microbiology Society. https://doi.org/10.1099/ijs.0.015016-0

Cox LM, Sohn J, Tyrrell KL, et al. Description of two novel members of the family Erysipelotrichaceae: Ileibacterium valens gen. nov., sp. nov. and Dubosiella newyorkensis, gen. nov., sp. nov., from the murine intestine, and emendation to the description of Faecalibaculum rodentium [published correction appears in Int J Syst Evol Microbiol. 2017 Oct;67(10):4289]. *Int J Syst Evol Microbiol*. 2017;67(5):1247-1254. doi:10.1099/ijsem.0.001793

Degelmann DM, Kolb S, Dumont M, Murrell JC, Drake HL. Enterobacteriaceae facilitate the anaerobic degradation of glucose by a forest soil. *FEMS Microbiol Ecol*. 2009;68(3):312-319. doi:10.1111/j.1574-6941.2009.00681.x

Garcia R, Stadler M, Gemperlein K, Müller R. *Aetherobacter fasciculatus* gen. nov., sp. nov. and *Aetherobacter rufus* sp. nov., novel myxobacteria with promising biotechnological applications. *Int J Syst Evol Microbiol*. 2016;66(2):928-938. doi:10.1099/ijsem.0.000813

Gerritsen, J., Hornung, B., Ritari, J., Paulin, L., Rijkers, G., Schaap, P., de Vos, W., & Smidt, H. (2019). A comparative and functional genomics analysis of the genus Romboutsia provides insight into adaptation to an intestinal lifestyle. *BioRxiv*, 845511. https://doi.org/10.1101/845511

Hardham JM, King KW, Dreier K, et al. Transfer of Bacteroides splanchnicus to Odoribacter gen. nov. as Odoribacter splanchnicus comb. nov., and description of Odoribacter denticanis sp. nov., isolated from the crevicular spaces of canine periodontitis patients. *Int J Syst Evol Microbiol*. 2008;58(Pt 1):103-109. doi:10.1099/ijs.0.63458-0

Iino, T., Mori, K., Tanaka, K., Suzuki, K. I., & Harayama, S. (2007). Oscillibacter valericigenes gen. nov., sp. nov., a valerate-producing anaerobic bacterium isolated from the alimentary canal of a Japanese corbicula clam. *International Journal of Systematic and Evolutionary Microbiology*, *57*(8), 1840–1845. https://doi.org/10.1099/ijs.0.64717-0

Jeong, H., Lim, Y. W., Yi, H., Sekiguchi, Y., Kamagata, Y., & Chun, J. (2007). Anaerosporobacter mobilis gen. nov., sp. nov., isolated from forest soil. *International Journal of Systematic and Evolutionary Microbiology*, *57*(8), 1784–1787. https://doi.org/10.1099/ijs.0.63283-0

Johnson DB, Stallwood B, Kimura S, Hallberg KB. Isolation and characterization of Acidicaldus organivorus, gen. nov., sp. nov.: a novel sulfur-oxidizing, ferric iron-reducing thermo-acidophilic heterotrophic Proteobacterium. *Arch Microbiol*. 2006;185(3):212-221. doi:10.1007/s00203-006-0087-7

Kanso, S., & Patel, B. K. C. (2003). Microvirga subterranea gen. nov., sp. nov., a moderate thermophile from a deep subsurface Australian thermal aquifer. *International Journal of Systematic and Evolutionary Microbiology*, *53*(2), 401–406. https://doi.org/10.1099/ijs.0.02348- 0

Kim, B. C., Jeon, B. S., Kim, S., Kim, H., Um, Y., & Sang, B. I. (2015). Caproiciproducens galactitolivorans gen. Nov., sp. nov., a bacterium capable of producing caproic acid from galactitol, isolated from a wastewater treatment plant. *International Journal of Systematic and Evolutionary Microbiology*, *65*(12), 4902–4908. https://doi.org/10.1099/ijsem.0.000665

Kläring, K., Hanske, L., Bui, N., Charrier, C., Blaut, M., Haller, D., Plugge, C. M., & Clavel, T. (2013). Intestinimonas butyriciproducens gen. nov., sp. nov., a butyrate-producing bacterium from the mouse intestine. *International Journal of Systematic and Evolutionary Microbiology*, *63*(PART 12), 4606–4612. https://doi.org/10.1099/ijs.0.051441-0

Krishnamurthi, S., Bhattacharya, A., Mayilraj, S., Saha, P., Schumann, P., & Chakrabarti, T. (2009). Description of Paenisporosarcina quisquiliarum gen. nov., sp. nov., and reclassification of Sporosarcina macmurdoensis Reddy et al. 2003 as Paenisporosarcina macmurdoensis comb. nov. *International Journal of Systematic and Evolutionary Microbiology*, *59*(6), 1364–1370. https://doi.org/10.1099/ijs.0.65130-0

Lagkouvardos, I., Lesker, T. R., Hitch, T. C. A., Gálvez, E. J. C., Smit, N., Neuhaus, K., Wang, J., Baines, J. F., Abt, B., Stecher, B., Overmann, J., Strowig, T., & Clavel, T. (2019). Sequence and cultivation study of Muribaculaceae reveals novel species, host preference, and functional potential of this yet undescribed family. *Microbiome*, *7*(1), 28. https://doi.org/10.1186/s40168- 019-0637-2

Lee H, Jung KB, Kwon O, et al. *Limosilactobacillus reuteri* DS0384 promotes intestinal epithelial maturation via the postbiotic effect in human intestinal organoids and infant mice. *Gut Microbes*. 2022;14(1):2121580. doi:10.1080/19490976.2022.2121580

Liu X, Mao B, Gu J, et al. *Blautia*-a new functional genus with potential probiotic properties?. *Gut Microbes*. 2021;13(1):1-21. doi:10.1080/19490976.2021.1875796

Mediannikov, O., Sekeyová, Z., Birg, M.-L., & Raoult, D. (2010). A Novel Obligate Intracellular Gamma-Proteobacterium Associated with Ixodid Ticks, Diplorickettsia massiliensis, Gen. Nov., Sp. Nov. *PLoS ONE*, *5*(7), e11478. https://doi.org/10.1371/journal.pone.0011478

Morotomi M, Nagai F, Watanabe Y. Description of Christensenella minuta gen. nov., sp. nov., isolated from human faeces, which forms a distinct branch in the order Clostridiales,

and proposal of Christensenellaceae fam. nov. *Int J Syst Evol Microbiol*. 2012;62(Pt 1):144-149. doi:10.1099/ijs.0.026989-0

Mozota M, Castro I, Gómez-Torres N, et al. Administration of *Ligilactobacillus salivarius* MP101 in an Elderly Nursing Home during the COVID-19 Pandemic: Immunological and Nutritional Impact. *Foods*. 2021;10(9):2149. Published 2021 Sep 11. doi:10.3390/foods10092149

Nagai, F., Morotomi, M., Sakon, H., & Tanaka, R. (2009). Parasutterella excrementihominis gen. nov., sp. nov., a member of the family Alcaligenaceae isolated from human faeces. *International Journal of Systematic and Evolutionary Microbiology*, *59*(7), 1793–1797. https://doi.org/10.1099/ijs.0.002519-0

Pfeiffer, N., Desmarchelier, C., Blaut, M., Daniel, H., Haller, D., & Clavel, T. (2012). Acetatifactor muris gen. nov., sp. nov., a novel bacterium isolated from the intestine of an obese mouse. *Archives of Microbiology*, *194*(11), 901–907. https://doi.org/10.1007/s00203-012-0822-1

Ricaboni D, Mailhe M, Cadoret F, Vitton V, Fournier PE, Raoult D. *'Colidextribacter massiliensis'* gen. nov., sp. nov., isolated from human right colon. *New Microbes New Infect*. 2016;17:27-29. Published 2016 Nov 28. doi:10.1016/j.nmni.2016.11.023

Rosero JA, Killer J, Sechovcová H, et al. Reclassification of *Eubacterium rectale* (Hauduroy *et al.* 1937) Prévot 1938 in a new genus *Agathobacter* gen. nov. as *Agathobacter rectalis* comb. nov., and description of *Agathobacter ruminis* sp. nov., isolated from the rumen contents of sheep and cows. *Int J Syst Evol Microbiol*. 2016;66(2):768-773. doi:10.1099/ijsem.0.000788

Sakamoto M, Benno Y. Reclassification of Bacteroides distasonis, Bacteroides goldsteinii and Bacteroides merdae as Parabacteroides distasonis gen. nov., comb. nov., Parabacteroides goldsteinii comb. nov. and Parabacteroides merdae comb. nov. *Int J Syst Evol Microbiol*. 2006;56(Pt 7):1599-1605. doi:10.1099/ijs.0.64192-0

Sarma-Rupavtarm RB, Ge Z, Schauer DB, Fox JG, Polz MF. Spatial distribution and stability of the eight microbial species of the altered schaedler flora in the mouse gastrointestinal tract. *Appl Environ Microbiol*. 2004;70(5):2791-2800. doi:10.1128/AEM.70.5.2791-2800.2004

Schnupf, P., Gaboriau-Routhiau, V., Gros, M., Friedman, R., Moya-Nilges, M., Nigro, G., Cerf- Bensussan, N., & Sansonetti, P. J. (2015). Growth and host interaction of mouse segmented filamentous bacteria in vitro. *Nature*, *520*(7545), 99–103. https://doi.org/10.1038/nature14027

Takeuchi, M., Hamana, K., & Hiraishi, A. (2001). Proposal of the genus Sphingomonas sensu stricto and three new genera, Sphingobium, Novosphingobium and Sphingopyxis, on the basis of phylogenetic and chemotaxonomic analyses. *International Journal of Systematic and Evolutionary Microbiology*, *51*(4), 1405–1417. https://doi.org/10.1099/00207713-51-4-1405

Tindall BJ. The names *Hungateiclostridium*Zhang *et al.* 2018, *Hungateiclostridium thermocellum* (Viljoen *et al.* 1926) Zhang *et al.*2018, *Hungateiclostridium cellulolyticum*(Patel *et al.* 1980) Zhang *et al.* 2018, *Hungateiclostridium aldrichii* (Yang *et al.* 1990) Zhang *et al.* 2018, *Hungateiclostridium alkalicellulosi* (Zhilina *et al.* 2006) Zhang *et al.*2018, *Hungateiclostridium clariflavum*(Shiratori *et al.* 2009) Zhang *et al.* 2018, *Hungateiclostridium straminisolvens* (Kato *et al.* 2004) Zhang *et al.* 2018 and *Hungateiclostridium saccincola* (Koeck *et al.*2016) Zhang *et al.* 2018 contravene Rule 51b of the International Code of Nomenclature of Prokaryotes and require replacement names in the genus *Acetivibrio* Patel *et al.* 1980. *Int J Syst Evol Microbiol*. 2019;69(12):3927-3932. doi:10.1099/ijsem.0.003685

Trachsel, J., Humphrey, S., & Allen, H. K. (2018). Butyricicoccus porcorum sp. nov., a butyrate- producing bacterium from swine intestinal tract. *International Journal of Systematic and Evolutionary Microbiology*, *68*(5), 1737–1742. https://doi.org/10.1099/ijsem.0.002738

Vandamme PA, Peeters C, Cnockaert M, et al. Bordetella bronchialis sp. nov., Bordetella flabilis sp. nov. and Bordetella sputigena sp. nov., isolated from human respiratory specimens, and reclassification of Achromobacter sediminum Zhang et al. 2014 as Verticia sediminum gen. nov., comb. nov. *Int J Syst Evol Microbiol*. 2015;65(10):3674-3682. doi:10.1099/ijsem.0.000473

Würdemann D, Tindall BJ, Pukall R, et al. Gordonibacter pamelaeae gen. nov., sp. nov., a new member of the Coriobacteriaceae isolated from a patient with Crohn's disease, and reclassification of Eggerthella hongkongensis Lau et al. 2006 as Paraeggerthella hongkongensis gen. nov., comb. nov. *Int J Syst Evol Microbiol*. 2009;59(Pt 6):1405-1415. doi:10.1099/ijs.0.005900-0

Yutin, N., & Galperin, M. Y. (2013). A genomic update on clostridial phylogeny: Gram-negative spore formers and other misplaced clostridia. *Environmental Microbiology*, *15*(10), 2631–2641. https://doi.org/10.1111/1462-2920.12173

Zheng J, Wittouck S, Salvetti E, et al. A taxonomic note on the genus *Lactobacillus*: Description of 23 novel genera, emended description of the genus *Lactobacillus*Beijerinck 1901, and union of *Lactobacillaceae*and *Leuconostocaceae*. *Int J Syst Evol Microbiol*. 2020;70(4):2782-2858. doi:10.1099/ijsem.0.004107
