# Supplementary material for: Early-life gut microbiota assembly patterns are conserved between laboratory and wild mice
Source: Commun Biol. 2024 Nov 7;7:1456. doi: 10.1038/s42003-024-07039-y (PMC11543677; doi:10.1038/s42003-024-07039-y)
Supplement: Supplementary file 9 — Reporting summary [file 42003_2024_7039_MOESM9_ESM.pdf]

Corresponding author(s): Eveliina Hanski
Sarah Knowles

Last updated by author(s): 20/8/2024

Reporting Summary

Nature Portfolio wishes to improve the reproducibility of the work that we publish. This form provides structure for consistency and transparency in reporting. For further information on Nature Portfolio policies, see our Editorial Policies and the Editorial Policy Checklist.

Statistics

For all statistical analyses, confirm that the following items are present in the figure legend, table legend, main text, or Methods section.

n/a	Confirmed	
☐	☒	The exact sample size (*n*) for each experimental group/condition, given as a discrete number and unit of measurement
☐	☒	A statement on whether measurements were taken from distinct samples or whether the same sample was measured repeatedly
☐	☒	The statistical test(s) used AND whether they are one- or two-sided<br>*Only common tests should be described solely by name; describe more complex techniques in the Methods section.*
☐	☒	A description of all covariates tested
☐	☒	A description of any assumptions or corrections, such as tests of normality and adjustment for multiple comparisons
☐	☒	A full description of the statistical parameters including central tendency (e.g. means) or other basic estimates (e.g. regression coefficient) AND variation (e.g. standard deviation) or associated estimates of uncertainty (e.g. confidence intervals)
☐	☒	For null hypothesis testing, the test statistic (e.g. *F*, *t*, *r*) with confidence intervals, effect sizes, degrees of freedom and *P* value noted<br>*Give P values as exact values whenever suitable.*
☒	☐	For Bayesian analysis, information on the choice of priors and Markov chain Monte Carlo settings
☒	☐	For hierarchical and complex designs, identification of the appropriate level for tests and full reporting of outcomes
☐	☒	Estimates of effect sizes (e.g. Cohen's *d*, Pearson's *r*), indicating how they were calculated

Our web collection on statistics for biologists contains articles on many of the points above.

Software and code

Policy information about availability of computer code

Data collection	No software used for data collection in this study.
Data analysis	Analysis was conducted using openly available R packages as described in Methods.

For manuscripts utilizing custom algorithms or software that are central to the research but not yet described in published literature, software must be made available to editors and reviewers. We strongly encourage code deposition in a community repository (e.g. GitHub). See the Nature Portfolio guidelines for submitting code & software for further information.

Data

Policy information about availability of data

All manuscripts must include a data availability statement. This statement should provide the following information, where applicable:

- Accession codes, unique identifiers, or web links for publicly available datasets
- A description of any restrictions on data availability
- For clinical datasets or third party data, please ensure that the statement adheres to our policy

All data used in this study is publicly available at GenBank under accession number PRJNA1028479 (https://www.ncbi.nlm.nih.gov/bioproject/1028479)

Research involving human participants, their data, or biological material

Policy information about studies with human participants or human data. See also policy information about sex, gender (identity/presentation), and sexual orientation and race, ethnicity and racism.

Reporting on sex and gender	N/A
Reporting on race, ethnicity, or other socially relevant groupings	N/A
Population characteristics	N/A
Recruitment	N/A
Ethics oversight	Animal work was carried under the UK Home Office project license PPL PB0178858 held at the University of Oxford and with a research permit from the Islands Conservation Advisory Committee and Natural Resources Wales.

Note that full information on the approval of the study protocol must also be provided in the manuscript.

Field-specific reporting

Please select the one below that is the best fit for your research. If you are not sure, read the appropriate sections before making your selection.

☐ Life sciences ☐ Behavioural & social sciences ☒ Ecological, evolutionary & environmental sciences

For a reference copy of the document with all sections, see nature.com/documents/nr-reporting-summary-flat.pdf

Ecological, evolutionary & environmental sciences study design

All studies must disclose on these points even when the disclosure is negative.

Study description	Lab and wild house mice were sampled to study early life dynamics in the gut microbiota. For this, 39 lab mice (C57BL/6) were sampled. 230 wild mice were live trapped and tagged with subcutaneous identification tags, allowing identification at re-capture (total of 433 samples from wild mice were included in this study). Faecal samples were used to characterise the gut microbiota using 16S rRNA amplicon sequencing approach. Alongside faecal sampling, various metadata were collected (e.g., sex, body weight) and used as covariates. These data were used to study whether lab and wild mice undergo similar early life gut microbial dynamics despite having distinct gut microbiotas per se.
Research sample	The subjects of this study were 39 lab and 230 wild mice sampled at the Biomedical Services Building, University of Oxford, and the Skokholm Island, Wales, respectively. Lab mice were sampled in October 2021. Wild mice were sampled in September-October 2021 and August-September 2020. Wild mice were trapped opportunistically using Sherman live traps. Data collected included mice of different age groups and sexes.
Sampling strategy	Lab mice were sampled opportunistically using mice at breeding facility. Lab mice were not subject to interventions at the time of study. Lab mice were sampled intentionally across mice of different age to capture age-related microbiota dynamics. Wild mice were trapped opportunistically across two sampling sites on the Skokholm Island house mouse study system. These sampling sites were established in 2019 and known to have dense populations of house mice, ensuring sampling success.
Data collection	Lab mice: Faecal samples were collected by placing mice on a sterile surface. Pellets were collected with sterile forceps, preserved in DNA/RNA Shield (Zymo Research, USA) and stored at -80°C until DNA extraction (≤12 months). Wild mice: Trapping was carried out in two sampling sites on the island (Fig. S1) using small Sherman traps provisioned with peanuts, non-absorbent cotton wool for bedding, and with a spray of sesame oil outside the trap used as a lure. On each trapping night, 150 traps were set at dusk and checked at dawn at one of the sampling sites. Visited traps (where signs of a mouse were detected, whether captured or not) were washed and sterilised with 20% bleach solution before re-use. Captured mice were tagged with a subcutaneous passive integrated transponder (PIT) tag for permanent identification, or identified through PIT tag detection upon recapture. All captures were therefore individually identified, sexed, scored for reproductive status, and weighed to the nearest 0.1g before release at their trapping point. Sex was determined based on measurement of anogenital distance. Faecal pellets were collected from traps, and preserved in DNA/RNA Shield. Samples were stored at -20°C during fieldwork (≤6 weeks), after which they were transported frozen to the laboratory and stored at -80°C until DNA extraction (≤17 months).
Timing and spatial scale	Lab mice were sampled at a single animal facility in October 2021. Wild mice were sampled across two sampling sites on the Skokholm Island, Wales, in September-October 2019 and August-September 2020.
Data exclusions	All collected lab mouse samples were included in analysis. For wild mice, female mice showing signs of pregnancy (bulging central body; n=15) were excluded, as these were associated with heavier body mass. We also excluded female mice ≥25g (n=65), with the

aim of excluding pregnant females that may not have been recognised as such. Applying these criteria resulted in a set of 433 faecal samples from 230 individual wild mice (1–10 samples each).

Reproducibility	Our overall findings on the differences between lab and wild mouse gut microbiota are highly similar to patterns found in other studies comparing the two. We have documented our methods at a level of detail that allows replication of our study (either with our data, which is publicly available, or with another study system). Lab protocols and computational methods used are publicly available.
Randomization	Faecal samples were randomised for DNA extraction by mixing all sample tubes together and then randomly selecting tubes. The samples were further randomised in the same manner for library preparation.
Blinding	This was an observational study using lab and wild individuals, hence blinding was not applicable.

Did the study involve field work? ☒ Yes ☐ No

Field work, collection and transport

Field conditions	Samples from wild mice were collected in Skokholm Island, Wales, UK. Temperatures ranged from +25 to +5 degrees celcius. Trapping was not conducted when two or more of the following weather conditions were forecast: (1) heavy rain (precipitation >7.6 mm per hour), (2) strong wind (>30 mph), (3) low temperature (<5.0c) for safety of fieldwork personnel as well as animal welfare reasons (e.g., minimisation of risk of hypothermia).
Location	Skokholm Island, Wales, UK (51.697, -5.278)
Access & import/export	Animal work was carried under the UK Home Office project license PPL PB0178858 held at the University of Oxford and with a research permit from the Islands Conservation Advisory Committee and Natural Resources Wales. Additionally, all field workers conducting licensed procedures (PIT tagging and ear marking) were trained and held personal licenses (PIL) for work with wild rodents.
Disturbance	It is expected that live trapping causes mild stress to the mice upon repeated trapping. This was minimised by releasing animals at point of capture as soon as possible as well as by providing nesting material and peanuts when trapped. Some mild trampling of ground vegetation was caused; however, movement on the island was strictly controlled to avoid disturbance to wildlife (e.g., seabirds).

Reporting for specific materials, systems and methods

We require information from authors about some types of materials, experimental systems and methods used in many studies. Here, indicate whether each material, system or method listed is relevant to your study. If you are not sure if a list item applies to your research, read the appropriate section before selecting a response.

Materials & experimental systems

n/a	Involved in the study	
☒	☐	Antibodies
☒	☐	Eukaryotic cell lines
☒	☐	Palaeontology and archaeology
☐	☒	Animals and other organisms
☒	☐	Clinical data
☒	☐	Dual use research of concern
☒	☐	Plants

Methods

n/a	Involved in the study	
☒	☐	ChIP-seq
☒	☐	Flow cytometry
☒	☐	MRI-based neuroimaging

Animals and other research organisms

Policy information about studies involving animals; ARRIVE guidelines recommended for reporting animal research, and Sex and Gender in Research

Laboratory animals	Study involved 39 lab mice (C57BL/6JOlaHsdOxuni) from the Biomedical Services Building, Oxford.
Wild animals	Wild mice were trapped with Sherman live traps. Traps were set at dusk and collected at dawn. Trapped mice were first checked for welfare concerns and prioritised accordingly (e.g., females appearing pregnant were released as soon as possible). Mice were tagged or identified at recapture, as well as subject to measurement of morphometrics, such as body length. After measurement, mice were released within 2m of trapping point.
Reporting on sex	Mice were sexed upon capture based on anogenital distance and appearance of genitalia. The wild mouse data included in the study (433 samples) includes >150 samples from each male and female mice. (Sex was listed as 'unknown' when it could not be clearly identified).

Field-collected samples	Faecal pellets were collected from traps, and preserved in DNA/RNA Shield. Samples were stored at -20°C during fieldwork (≤6 weeks), after which they were transported frozen to the laboratory and stored at -80°C until DNA extraction (≤17 months).
Ethics oversight	Animal work was carried under the UK Home Office project license PPL PB0178858 held at the University of Oxford and with a research permit from the Islands Conservation Advisory Committee and Natural Resources Wales. Additionally, all field workers conducting licensed procedures (PIT tagging and ear marking) were trained and held personal licenses (PIL) for work with wild rodents.

Note that full information on the approval of the study protocol must also be provided in the manuscript.

Plants

Seed stocks	*Report on the source of all seed stocks or other plant material used. If applicable, state the seed stock centre and catalogue number. If plant specimens were collected from the field, describe the collection location, date and sampling procedures.*
Novel plant genotypes	*Describe the methods by which all novel plant genotypes were produced. This includes those generated by transgenic approaches, gene editing, chemical/radiation-based mutagenesis and hybridization. For transgenic lines, describe the transformation method, the number of independent lines analyzed and the generation upon which experiments were performed. For gene-edited lines, describe the editor used, the endogenous sequence targeted for editing, the targeting guide RNA sequence (if applicable) and how the editor was applied.*
Authentication	*Describe any authentication procedures for each seed stock used or novel genotype generated. Describe any experiments used to assess the effect of a mutation and, where applicable, how potential secondary effects (e.g. second site T-DNA insertions, mosiacism, off-target gene editing) were examined.*
